# Biomarkers associated with post-operative pneumonia: a systematic review and meta-analysis

**DOI:** 10.1186/s12890-026-04486-7

**Published:** 2026-07-16

**Authors:** Fiona Howroyd, Amanda Veiga Sardeli, Fang Gao Smith, Tonny Veenith, Niharika A. Duggal, Zubair Ahmed

**Affiliations:** 1 Department of Inflammation and Ageing, School of Infection, Inflammation and Immunology, College of Medicine and Health, University of Birmingham, Birmingham, UK; 2 University Hospitals Birmingham NHS Foundation Trust, Birmingham, UK; 3 Royal Wolverhampton Hospital, Wolverhampton, UK; 4 Institute of Acute Care, University of Wolverhampton, Wolverhampton, UK

**Keywords:** Biomarkers, Pneumonia, Pulmonary complications, Respiratory infection, Surgery

## Abstract

**Background:**

Post-operative pneumonia is a commonly occurring surgical complication associated with poor patient outcomes. This study aimed to synthesise pre-operative and post-operative blood-based biomarkers associated with post-operative pneumonia.

**Methods:**

Electronic databases were searched up to April 14th, 2026. Primary studies investigating blood-based biomarkers in adults hospitalised after surgery were included. Meta-analysis was performed using the random effects model to compare pooled data for pneumonia and no-pneumonia groups using standardised mean difference. Risk of bias was assessed using the ROBINS-E tool.

**Results:**

Thirty-seven studies (*n* = 15,842 patients) were included, with an overall pneumonia rate of 17.8% [15.8–20.0%]. One hundred and fifteen biomarkers were identified. Meta-analysis identified that patients who developed post-operative pneumonia had significantly lower platelet-to-neutrophil ratio (*p* < 0.0001), red blood cell count (*p* = 0.001), haemoglobin (*p* = 0.002), albumin (*p* = 0.005) and lymphocyte count (*p* = 0.008) and significantly higher monocyte-to-lymphocyte ratio (*p* < 0.0001), systemic immune inflammation index (*p* < 0.001), systemic inflammatory response index (*p* < 0.0001) and blood urea nitrogen (*p* = 0.0001) at pre-operative baseline, compared to those without pneumonia. Patients who developed post-operative pneumonia were associated with significantly higher procalcitonin (PCT) at post-operative day one (*p* = 0.03), two (*p* = 0.0001), three (*p* = 0.0004) and six (*p* < 0.0001); higher C-Reactive Protein (CRP) at day two (*p* = 0.02), four (*p* < 0.0001) and five (*p* < 0.0001); higher interleukin (IL)-6 at day three (*p* = 0.001) and four (*p* = 0.0002); and higher white blood cell (WBC) count at day three (*p* = 0.01) and day four (*p* = 0.001) post-operatively, compared to those without pneumonia. In addition, pre-operative CRP was associated with mortality within the patients who later developed post-operative pneumonia (*r* = 0.70, *p* = 0.0009).

**Conclusions:**

The results identify blood-based biomarker signals associated with post-operative pneumonia. However, interpretation is limited by heterogeneous pneumonia definitions and inconsistent reporting of diagnosis timing across studies. The certainty of evidence is therefore limited, and thus these biomarkers cannot currently be used for prediction or diagnosis in clinical practice. Further high‑quality, prospective studies are required to establish clinically meaningful thresholds.

**Protocol registration:**

PROSPERO: CRD42024570654.

**Graphical Abstract:**

**Figure:**
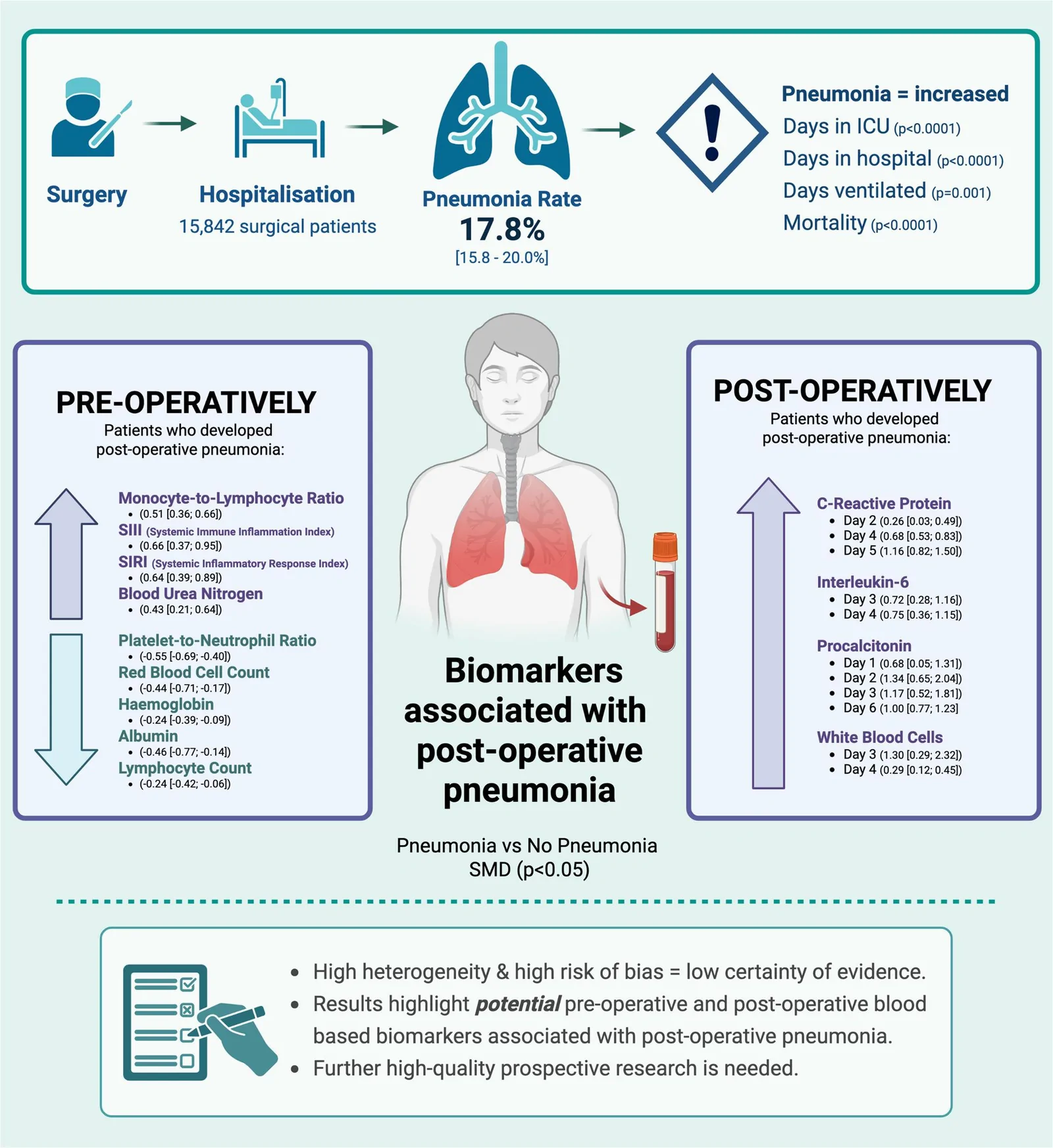
Biomarkers associated with post-operative pneumonia.

**Supplementary Information:**

The online version contains supplementary material available at https://doi.org/10.1186/s12890-026-04486-7.

## Introduction

Post-operative pneumonia (POP) is a common and serious complication following surgery [1]. POP is defined as a pulmonary infection occurring after surgery, typically occurring within the early post-operative period, although specific temporal definitions vary between studies [1, 2]. As the most commonly occurring post-surgical complication, POP is associated with poor prognosis, with increased in-hospital mortality rates and lower five-year survival [3]. Furthermore, POP increases the duration of mechanical ventilation and intensive care unit (ICU) and hospital admission, subsequently increasing healthcare costs [1]. With over 11 million surgical procedures performed annually by the National Health Service (NHS) in the UK, surgical morbidity and mortality are a significant public health issue due to their impact on patient outcomes and NHS resource utilisation [4].

Early and accurate identification of POP is critical to improving patient outcomes; however, clinical signs are often non-specific in the post-operative setting, where inflammation, pain, and physiological stress responses can mimic infection. The pathogenesis of POP is also complex, with growing prevalence of polymicrobial and antimicrobial resistance strains, subsequently making intervention and treatment increasingly challenging [1]. POP usually manifests due to bacterial colonisation of the aero-digestive tract, aspiration of contaminated secretions and impaired host-defences due to the impact of anaesthesia, critical illness and pre-morbid status [1]. However, the clinical symptoms of POP are broad and non-specific, encapsulating a breadth of infective and non-infective pathologies such as atelectasis or acute respiratory distress syndrome, that are subsequently grouped together as post-operative pulmonary complications (PPCs) [5]. Conventional investigative tools, including radiographic findings and microbiological cultures, are frequently delayed, insensitive, or difficult to interpret. Identifying POP in clinical practice is therefore challenging. Objective and specific methods to improve early identification of POP are therefore urgently required, to enhance prevention and treatment strategies with appropriate antibiotic use, and to ultimately improve patient outcomes [5].

Biomarkers may offer the potential for early, standardised and quantifiable identification of POP. A biomarker is a defined measure of a biological process, that can be used to detect the susceptibility or presence of disease [6]. As such, biomarkers may offer the potential to improve the identification of POP and subsequently reduce antibiotic prescription pressures, morbidity, mortality and the health economics associated with surgical complications. Although a promising prospect, the most effective biomarker for POP is currently unknown. To date, there is no comprehensive synthesis of the evidence evaluating biomarkers specifically for POP. A systematic review is therefore needed to critically appraise and integrate existing studies, identify the most promising biomarkers, assess their accuracy and clinical applicability, and to highlight key gaps for future research.

A related systematic review has examined blood-based biomarkers in the context of major trauma; however, surgical populations represent a distinct physiological context, characterised by controlled tissue injury and often have the availability of pre-operative baseline measurements [7]. This provides a unique opportunity to evaluate peri-operative changes in biomarker profiles and their association with the development of POP. Consequently, a focused synthesis of biomarker performance in surgical patients is warranted. Such a synthesis has the potential to inform clinical decision-making, guide the design of prospective studies, and ultimately improve surgical patient outcomes through earlier and more targeted intervention. This systematic review therefore aimed to:

1. Identify pre-operative blood-based biomarkers associated with the development of POP.
2. Identify post-operative blood-based biomarkers associated with the development of POP.
3. Identify associations between POP biomarkers and prognostic outcomes.

## Materials and methods

This systematic review was reported following the Preferred Reporting Items for Systematic Review and Meta-Analysis (PRISMA) guidelines (Additional File 1) [8]. The protocol for this systematic review and meta-analysis was registered with the international prospective register of systematic reviews (PROSPERO CRD42024570654) [9].

Since protocol publication several deviations have been made, in line with evolving evidence and methodological considerations. Firstly, the scope of the review was broadened to include biomarkers associated with POP in both the pre-operative and post-operative period, rather than specifically for diagnosis. This change was made due to heterogeneity in clinical diagnostic criteria across studies, which limited consistent classification of diagnostic performance. Second, subgroup analyses were refined according to the timing of biomarker measurement (pre-operative and post-operative), to better account for temporal variability in biomarkers. Third, the literature search dates were updated to ensure inclusion of the most recent evidence. Fourth, the definition of adults was standardised as individuals aged ≥ 18 years to ensure consistency across included studies. Fifth, statistical analyses were conducted using Biostat’s Comprehensive Meta-Analysis software (version 3.0; New Jersey, USA), selected for its advanced meta-analytic functionality and availability. Sixth, meta-analyses were undertaken where two or more studies were available, rather than the three studies specified in the protocol, to maximise data synthesis in the context of a limited evidence base. Finally, additional meta-regression analyses were performed to explore the prognostic effects of significant biomarkers, to contextualise the clinical relevance and potential utility of biomarkers associated with post-operative pneumonia. These modifications were made prior to or during data analysis and were not expected to substantially alter the overall conclusions, although they may have introduced some additional bias.

### Search strategy

This search was conducted across four electronic databases including PubMed, Cochrane Library, Ovid Medline and EMBASE. Published studies from all years, up to April 14th, 2026, were identified using a combination of synonyms for surgery, pneumonia and biomarkers within the title, abstract and MeSH terms where available, as detailed in Additional File 2.

The search strategy was designed to maximise sensitivity in order to capture all potentially relevant studies, given the variability in terminology used to describe post-operative pneumonia across the literature. As pneumonia is frequently reported within broader categories such as post-operative, pulmonary, or infective complications, both specific and broader terms were included. Similarly, broad surgical terms were used to reflect the wide range of surgical procedures across specialties and international settings, where terminology and indexing may vary. Free-text terms were applied to title and abstract fields to maximise sensitivity, alongside the use of controlled vocabulary (such as MeSH) to ensure comprehensive indexing. The strategy was adapted for each database to ensure appropriate use of indexing (Additional File 2).

### Selection process

Duplicate studies were removed prior to screening of the title and abstract by two independent, blinded reviewers (FH and ZA) using the software, Rayyan [10]. The full texts of the shortlisted studies were then assessed for eligibility. Any disagreements were resolved by discussion to reach consensus.

### Eligibility criteria

This systematic review included full-text, primary articles published in the English language that identified blood-based biomarkers for POP. There was no limit to the time, location or type of clinical study published. The inclusion criteria consisted of; (1) human studies (2), hospitalised adults (aged $\:\ge\:$18 years) (3), undergoing any type of surgery (4), assessed for POP via any clinical criteria (5), investigated for any blood-based biomarker, by any method and at any time point (6), reporting biomarker data for pneumonia and no-pneumonia groups. The exclusion criteria comprised of; (1) studies not available in full-text in the English language; (2) non-original studies or grey literature [including: case studies, narrative or systematic reviews, editorials, book chapters, conference abstracts, unpublished thesis or dissertations, study protocols]; (3) animal studies; (4) paediatric studies [population aged <18 years]; (5) studies that did not specify pneumonia as the post-operative complication; (6) studies without a no-pneumonia control group.

### Data collection process including data items

Data extraction was completed by one reviewer (FH) and checked by a second reviewer (ZA) using predesigned forms in Microsoft Excel. Any discrepancies in data extraction were resolved through discussion. The key characteristics of each included study was collated, including; author names, year of publication, location of the study, type of surgery, pneumonia frequency, the method used to diagnose pneumonia and the biomarkers reported, including the timepoint and method of assessment. The types of surgery were grouped into sub-categories, including; cardiothoracic, general, orthopaedic, neurosurgery and mixed. Pneumonia was recorded as either hospital-acquired pneumonia (HAP), ventilator-associated pneumonia (VAP) or aspiration pneumonia. The time point of biomarker assessment was grouped into pre-operative or post-operative, as recorded in each respective study. Post-operative timepoints were standardised to hours when less than 24-hours, or days when over 24-hours. Age, gender and prognostic outcomes including mortality, ICU and hospital length of hospital stay and days ventilated were recorded for sub-group analysis. For the main analysis, mean, standard deviation (SD) and sample size were collected for each potential biomarker in pneumonia and no-pneumonia groups. If biomarker data was presented as median and interquartile range (IQR), data was converted to mean and SD (SD = IQR / 1.35) [11]. However, we recognise that this approach assumes approximate normality and may be less reliable for skewed biomarkers. In the event of missing data, authors were contacted directly via email to request for mean, SD and sample size data. If authors did not reply within two months, then the study was excluded due to missing data.

### Risk of bias assessment

As the literature search generated observational studies, the risk of bias assessment was conducted using the ROBINS-E tool [12]. Risk of bias against the seven domains, as well as the overall risk of bias, were judged from low risk to very high risk by two authors independently (FH and ZA). Any discrepancies were resolved through discussion.

### Statistical analysis

The pooled rates of pneumonia were analysed using Biostat’s Comprehensive Meta-Analysis software (version 3.0; New Jersey, USA), employing the continuous data function and random effects model. A complementary exploratory analysis of study-level pneumonia rates was additionally performed using GraphPad PRISM^®^ software; comparing mean pneumonia rates across surgery and pneumonia subtypes using one-way analysis of variance (ANOVA) with Bonferroni correction. This analysis was intended to assess broad between-group patterns and was not designed to provide pooled inferential estimates equivalent to formal meta-analysis. Descriptive data of patient demographics were reported as mean and SD.

Meta-analysis of pooled biomarker data was conducted using Biostat’s Comprehensive Meta-Analysis software as an exploratory analysis of study-level effect sizes to assess timepoint variation in between-group differences. Given the heterogeneity in case‑mix and pneumonia definitions, the analyses are not intended to support clinical inference. Pooled biomarker data from the included studies was analysed using the continuous data function and employing a random effects model. Analyses were conducted separately for pre- and post-operative timepoints, with post-operative biomarkers further grouped by post-operative day, thereby allowing multiple measurements per study at different biomarker timepoints. The standardised mean difference (SMD), 95% confidence intervals (CI) and p values for the test of the overall effect, along with Q and I^2^ statistics were recorded for each biomarker when reported in two or more studies. For biomarkers reported in one study, the SMD and 95% CI were calculated.

Assessment of small‑study effects/publication bias using the Egger test was restricted to biomarkers with sufficient numbers of studies (i.e. ten or more studies) to make such tests interpretable; where the number of studies was small, these tests were not emphasised. The pooled mean values of statistically significant pre-operative biomarkers were displayed using GraphPad PRISM^®^ software to provide an exploratory observation of clinically meaningful metrics. Meta-regression analyses were performed for biomarkers with more than one recorded time-point to evaluate the linear association of biomarkers over time.

Sensitivity analyses were conducted for statistically significant meta-analysed pre- and post-operative biomarkers, and where sufficient data were available. Meta-analyses were repeated, as described above, but after: (1) exclusion of studies judged to be at high or very high risk of bias according to the ROBINS-E tool; and (2) exclusion of studies requiring conversion of summary statistics to estimated means and standard deviations. Sensitivity analyses were performed only when two or more studies remained following application of these exclusions. Results were compared with the primary analyses with respect to effect size magnitude, direction, statistical significance and heterogeneity. Due to low study numbers, leave-one-out sensitivity analyses were not performed because most biomarker meta-analyses were based on a small number of studies. In this context, removal of a single study results in disproportionate changes in the pooled estimate and variance structure, often reducing the analysis to very few studies (or a single study), thereby limiting interpretability and increasing the risk of spurious conclusions. Accordingly, leave-one-out approaches were considered methodologically uninformative.

Prognostic outcomes of pneumonia and no-pneumonia groups were analysed using the continuous data function and employing a random effects model, reporting the raw mean difference (RMD), 95% CI and p values for the test of overall effect when reporting number of days, and Odds Ratio (OR) for mortality. We also ran meta-regression analyses to evaluate the prognostic effect of significant biomarkers. A p value ≤ 0.05 was deemed as statistically significant throughout.

Since a large number of biomarkers and post-operative timepoints were evaluated, no formal adjustment for multiple comparisons were applied. All reported p-values are nominal. As a result, the threshold for statistical significance (*p* ≤ 0.05) should therefore be interpreted with caution, as some significant associations may represent type 1 errors (i.e. false positives) arising from multiple testing.

In addition, pooling of diagnostic accuracy measures (e.g. ROC/AUC) was explored; however, this was not feasible due to insufficient and inconsistent reporting across studies. Therefore, this systematic review was not designed to evaluate diagnostic performance, and findings should not be interpreted as evidence of predictive or diagnostic accuracy. Given the extent of clinical heterogeneity, analyses were intended to explore associations across heterogeneous contexts rather than to establish population‑wide or surgery‑specific biomarker effects.

## Results

**Fig. 1:**
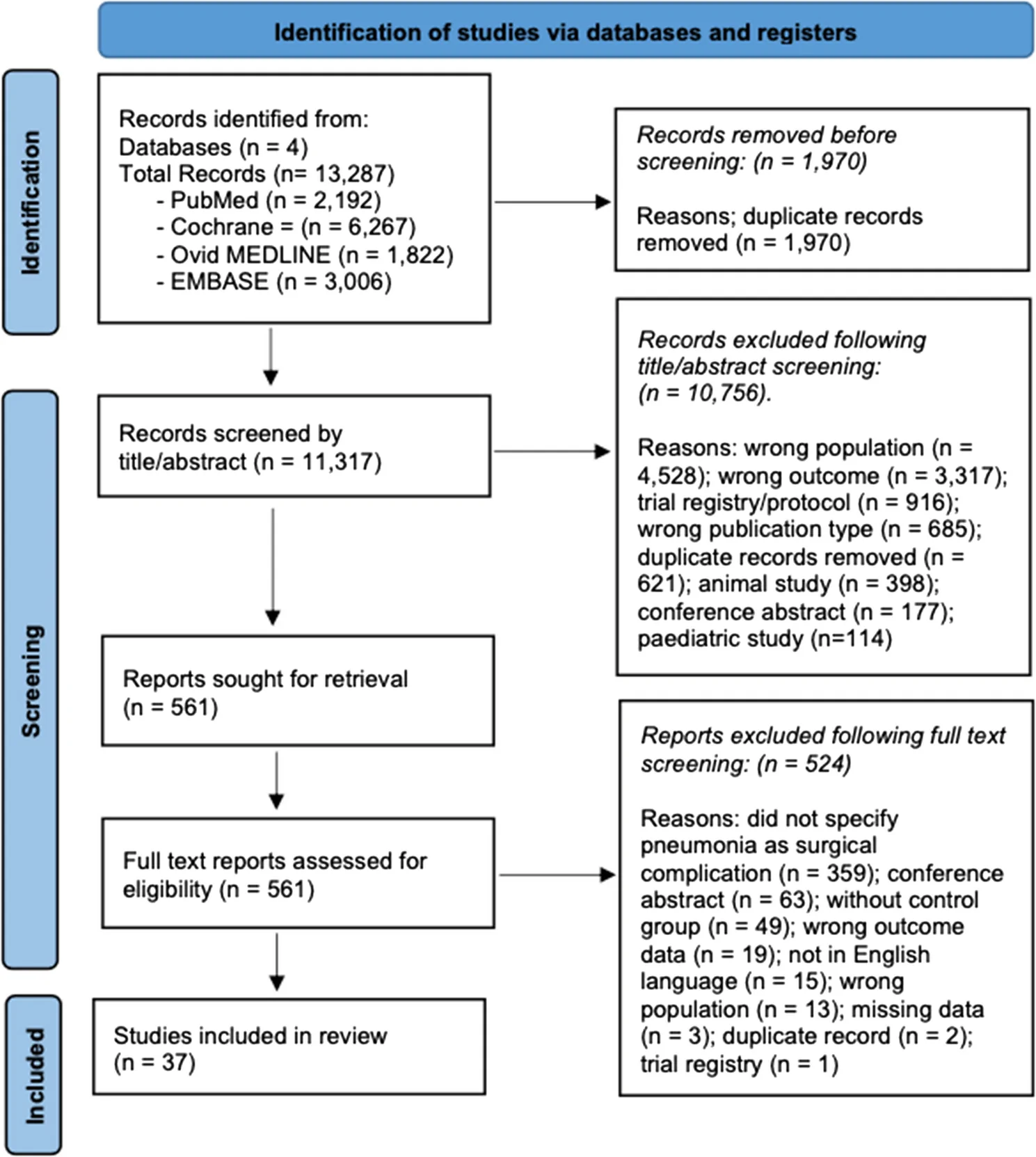
PRISMA flow chart of studies selection

The study selection and screening process is presented in Fig. 1. Our search retrieved 13,287 articles from four electronic databases. After initial screening, 561 studies remained for full text review, with 37 studies included in the final analysis. A summary of the key characteristics of the 37 included studies are detailed in Table 1 [13–49]. There was a total of 15,842 surgical patients of whom 51.06% (*n* = 8089) were female, with an overall mean age of 65.01 ± 12.44 years. Five surgical subtypes were identified across the studies, including cardiothoracic (17 studies, *n* = 4595 patients), general surgery (8 studies, *n* = 2052 patients), trauma and orthopaedic (T&O) (7 studies, 5931 patients), neurosurgery (4 studies, *n* = 2766 patients) and mixed (1 study, *n* = 498 patients).

**Table 1:** Study characteristics of included studies

Author	Origin	Surgery	Study Design	Sample Size	Age (years)	Sex (Male/Female)	Pneumonia Sub-Type	Pneumonia Rate (%)
Cardiothoracic Surgery								
As, 2025 [37]	Turkey	Cardiac with CPB	Retrospective observational	549	58.65 ± 7.91	367 M / 182 F	HAP	71 (12.93)
Li J, 2025 [41]	China	Cardiac	Prospective observational	537	66.1 ± 7.6	395 M / 142 F	HAP	66 (12.3)
Mao, 2025 [43]	China	Cardiac with CPB	Retrospective case-control	220	NR	141 M / 79 F	HAP	56 (25.45)
Ruan, 2025 [45]	China	Lung resection	Retrospective observational	252	63.89 ± 11.43	157 M / 95 F	HAP	66 (26.2)
Averdunk, 2020 [13]	Germany	Aneurysm repair (tAAA)	Prospective observational	33	63 ± 16.2	16 M / 17 F	HAP	6 (18.18)
Chen, 2022 [14]	China	Cardiac with CPB	Prospective observational	694	57.89 ± 13.32	410 M / 284 F	HAP	68 (9.8)
de la Varga Martinez, 2022 [15]	Spain	Cardiac valve	Prospective observational	803	70.13 ± 10.28	387 M / 416 F	HAP	42 (5.23)
Desmard, 2015 [16]	France	Lung transplant	Prospective observational	26	52 ± 27.45	17 M / 9 F	HAP	10 (38.46)
Jiao, 2015 [17]	China	Cardiac with CPB	Prospective observational	92	48.08 ± 11.79	47 M / 45 F	VAP	33 (35.87)
Jin, 2021 [18]	China	Cardiac with CPB	Prospective observational	272	59.77 ± 11.93	153 M / 119 F	HAP	24 (8.82)
Li, 2021 [19]	China	Off-pump CABG	Retrospective observational	131	61.1 ± 8.2	98 M / 33 F	HAP	23 (17.56)
Liu, 2015 [20]	China	Cardiac with CPB	Retrospective observational	17	47.66 ± 5.73	6 M / 11 F	VAP	7 (41.18)
Liu, 2019 [21]	China	Off-pump CABG	Retrospective observational	402	64.71 ± 8.72	314 M / 88 F	HAP	44 (10.95)
Perrotti, 2018 [22]	France	Elective cardiac	Prospective observational	155	69.8 ± 9.2	112 M / 43 F	HAP	17 (10.97)
Song, 2020 [23]	China	Cardiac valve	Retrospective observational	80	43.94 ± 10.95	49 M / 31 F	VAP	40 (50)
White, 2011 [24]	Ireland	Lung resection	Prospective observational	60	64.25 ± 1.57	42 M / 18 F	HAP	19 (31.67)
Xie, 2022 [25]	China	Cardiac with CPB	Prospective observational	272	59.76 ± 11.66	153 M / 119 F	HAP	24 (8.82)
General Surgery								
Ding, 2025 [38]	China	Oesophagectomy	Retrospective observational	312	66 ± 6.6	171 M / 141 F	HAP	86 (27.6)
Ishida, 2024 [39]	Japan	Oesophagectomy	Prospective observational	439	68 ± 8.17	342 M / 97 F	HAP	96 (21.9)
Lin, 2024 [42]	China	Abdominal surgery	Retrospective observational	265	68 ± 4.93	134 M / 141 F	VAP	93 (35.1)
Ozawa, 2026 [44]	Japan	Oesophagectomy	Prospective observational	63	68.5 ± 8.3	49 M / 14 F	HAP	11 (17.5)
Wen, 2025 [47]	China	Laparoscopic abdominal	Prospective observational	356	NR	179 M / 177 F	HAP	112 (31.46)
Zhou, 2024 [49]	China	Kidney transplant	Case control study	93	41.33 ± 11.55	64 M / 29 F	HAP	31 (33.3)
Singh, 2023 [34]	India	Elective abdominal	Prospective observational	200	47.38 ± 12.01	128 M / 72 F	HAP	28 (14)
Toh, Yoon, 2019 [35]	Japan	PEG	Retrospective observational	324	82.3 ± 10.9	149 M / 175 F	Aspiration	72 (22.22)
Trauma & Orthopaedic Surgery								
Li F, 2025 [40]	China	Femoral fracture	Retrospective cohort	193	82.9 ± 9.05	75 M / 118 F	HAP	49 (25.4)
Tang, 2024 [46]	China	Hip fracture	Retrospective cohort	1279	74.7 ± 9.55	508 M / 771 F	HAP	117 (9.1)
Wu, 2025 [48]	China	Hip fracture	Retrospective observational	637	81.43 ± 7.48	323 M / 314 F	HAP	61 (9.58)
Ji, 2021 [26]	China	Hip fracture	Retrospective observational	901	78.69 ± 7.11	303 M / 598 F	HAP	55 (6.1)
Lv, 2016 [27]	China	Hip fracture	Prospective observational	1429	75 ± 12.59	597 M / 832 F	HAP	70 (4.9)
Wang, 2020 [28]	China	Hip fracture	Retrospective observational	293	85.1 ± 3.3	96 M / 197 F	HAP	33 (11.26)
Yao, 2023 [29]	China	Hip fracture	Retrospective observational	1199	74.77 ± 9.61	479 M / 720 F	HAP	111 (9.26)
Neurosurgery								
Yuan, 2022 [30]	China	aSAH	Retrospective observational	843	54.7 ± 11.1	352 M / 491 F	HAP	257 (30.49)
Zhang, 2022 [31]	China	aSAH	Retrospective observational	395	60.19 ± 10.22	121 M / 274 F	HAP	78 (19.75)
Zhao, 2022 [32]	China	Craniotomy	Retrospective observational	372	48 ± 13	161 M / 211 F	HAP	18 (4.84)
Zuo, 2019 [33]	China	Meningioma Resection	Retrospective observational	1156	52.06 ± 13.41	340 M / 816 F	HAP	51 (4.41)
Mixed Surgical								
Chen, 2023 [36]	China	Mixed surgical	Prospective observational	498	71.8 ± 5.2	328 M / 170 F	HAP	84 (16.8)

Age: documented as mean ± SD; Sex: *n* M = male / *n* F = female; Pneumonia rate *n* (%)

*Abbreviations*: *aSAH *aneurysmal subarachnoid haemorrhage, *CABG *coronary artery bypass surgery, *CPB *cardiopulmonary bypass, *HAP *hospital acquired pneumonia, *PEG *percutaneous endoscopic gastrostomy, *tAAA *thoracoabdominal aortic aneurysm repair, *VAP *ventilator associated pneumonia

### Risk of bias analysis

**Fig. 2:**
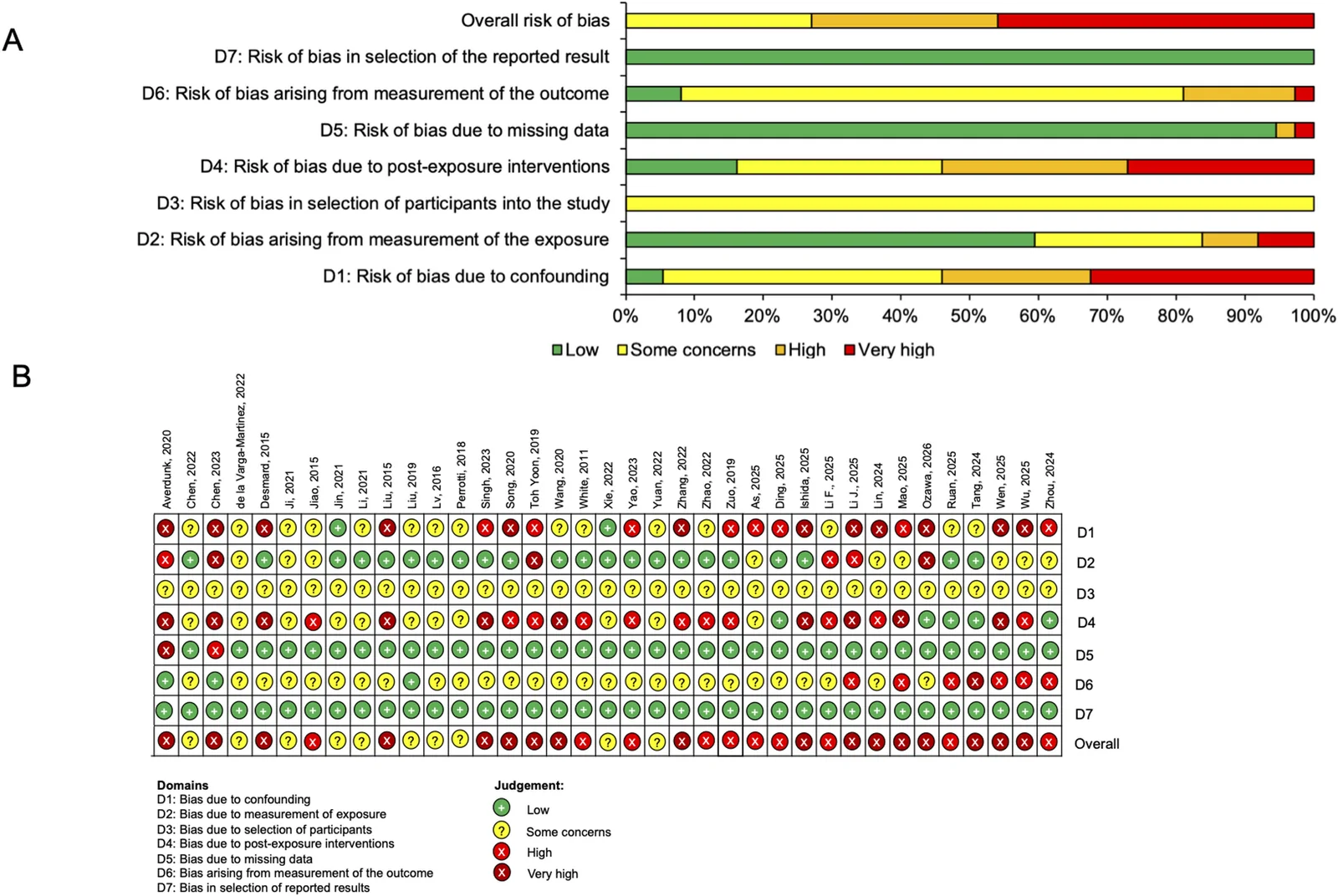
Risk of bias assessment of included studies [13–49]. **A** Summary chart to show the risk of bias across the 7 domains in all studies; (**B**) Diagram to show risk of bias in individual studies for each of the 7 domains

The risk of bias analysis demonstrated ratings of some concerns to very high risk of bias in all of the included studies; with 45.9% of the studies judged as having very high overall risk of bias (Fig. 2a). Over half of the studies had a high or very high risk of bias due to confounding (21.6% and 32.4% respectively) and due to post-exposure interventions (27.0% and 27.0% respectively) (Fig. 2b).

### Pneumonia rates & clinical diagnosis

**Fig. 3:**
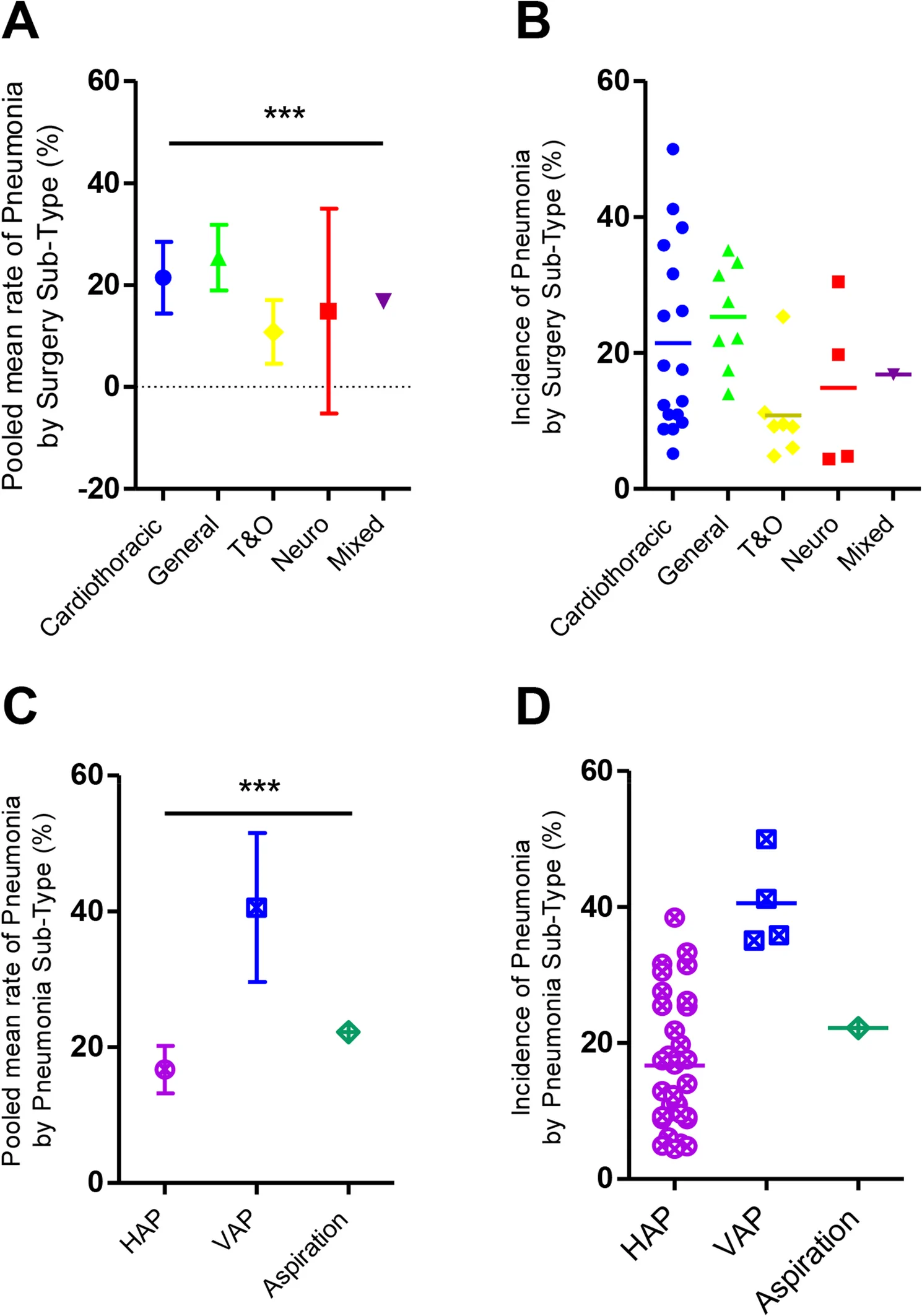
Incidence rates of pneumonia categorised by surgery and pneumonia subtypes. **A** Pooled mean rates of pneumonia by surgery subtype; (**B**) Mean values of pneumonia rates reported by individual studies, according to surgery subtype; (**C**) Pooled mean rates of pneumonia by pneumonia subtype; (**D**) Mean values of pneumonia rates reported by individual studies, according to pneumonia subtype. Note: Fig. 3B and D demonstrate a complementary analysis and should be interpreted with caution as the studies are not weighted. Note: only one study reported incidence rates in the mixed surgical subgroup and the aspiration pneumonia subgroup. Key: VAP = ventilator-associated pneumonia; HAP: hospital acquired pneumonia; T&O = trauma and orthopaedic surgery; Neuro = neurosurgical. *** = *p* < 0.001

The overall pooled incidence rate of pneumonia was 17.8% (15.8–20.0%). There was a statistically significant difference in pneumonia incidence rates between surgical subtypes (*p* < 0.00001) (Fig. 3A&B) and pneumonia subtypes (*p* < 0.0001) (Fig. 3C&D). The general surgery subgroup had the highest incidence of pneumonia across all surgical subgroups, with a pooled mean pneumonia rate of 25.20% [20.7–30.4%], followed by cardiothoracic surgery (17.9% [13.2–23.9%]). In the one study reporting mixed surgical cohorts the pooled incidence rates of pneumonia was 16.9% [13.8–20.4%], whilst after neurosurgery rates were 11.3% [4.0–28.2%] and T&O surgery 9.6% [6.8–13.4%]. The majority of studies reported pneumonia as HAP (32 studies), with four studies reporting VAP and one study aspiration pneumonia. The pooled mean rates of VAP (39.5% [32.4–47.1%]) were higher than the pooled mean rates of aspiration pneumonia (22.2% [18.0–27.1%]) and HAP (14.3% [11.4–17.9%]).

The pneumonia diagnosis methods varied between studies using a range of clinical, radiological and microbiological methods and reference standards (Additional File 3). Although 21 studies referenced national guideline frameworks, ten different guideline definitions were used. Eighteen studies pre-defined their pneumonia assessment period, ranging from day two to 30 post-operatively in fourteen of the studies, with four studies monitoring for pneumonia until discharge from ICU or hospital. Only six studies reported the time of confirmed pneumonia diagnosis (ranging from day two to 21 after surgery). This variability indicates substantial heterogeneity in outcome definition across the included evidence base.

### Biomarker meta-analysis

A total of 115 different blood-based biomarkers were identified. There were 110 biomarkers which underwent pre-operative assessment, with 48 reported in two or more studies and therefore feasible for meta-analysis (Table 2) and 62 biomarkers in one study only (Additional File 4). Thirty-four of the biomarkers underwent post-operative assessment, with six reported in two or more studies (Table 3) and 28 in one study only (Additional File 5). None of the included studies provided sufficient and comparable diagnostic accuracy data to permit pooled ROC or AUC analyses.

**Table 2:** Pre-operative biomarkers in$\:\ge\:$2 studies

Biomarker	SMD	LL	UL	*p*	Sample Size (*n*)		K	I^2^%	Q	Tau^2^
					Pop	No-POP				
Platelet-to-neutrophil ratio	-0.545	-0.69	-0.40	**< 0.0001**	257	586	2	0.00	0.97	0.00
Monocyte-to-lymphocyte ratio	0.510	0.36	0.66	**< 0.0001**	257	586	2	0.00	0.25	0.00
Systemic immune inflammation index (SII)	0.659	0.37	0.95	**< 0.0001**	324	1858	5	77.20	17.54	0.08
Systemic inflammatory response index (SIRI)	0.636	0.39	0.89	**< 0.0001**	257	586	2	63.64	2.75	0.02
Blood urea nitrogen	0.425	0.21	0.64	**0.0001**	231	1836	4	56.06	6.83	0.03
Red blood cell count	-0.443	-0.71	-0.17	**0.001**	148	1224	2	35.33	1.55	0.02
Haemoglobin (Hb)	-0.240	-0.39	-0.09	**0.002**	536	6285	10	61.69	23.50	0.04
Albumin	-0.455	-0.77	-0.14	**0.005**	560	4978	9	91.44	93.49	0.21
Lymphocyte count	-0.244	-0.42	-0.06	**0.008**	783	4758	9	79.86	39.73	0.06
Platelet-to-lymphocyte ratio	0.370	-0.01	0.75	0.05	126	1388	3	65.58	5.81	0.07
Leukocyte count	-0.296	-0.61	0.01	0.06	50	398	2	8.05	1.09	0.00
C-Reactive Protein (CRP)	0.215	-0.01	0.44	0.07	391	2850	9	75.00	32.00	0.09
D-Dimer	0.357	-0.04	0.75	0.08	226	1541	3	83.79	12.34	0.10
Sodium	-0.130	-0.28	0.02	0.09	223	2245	4	7.10	3.23	0.00
Calcium	-0.292	-0.63	0.05	0.09	190	1985	3	73.35	7.50	0.06
Globulin	0.135	-0.03	0.30	0.11	199	2329	3	12.04	2.27	0.00
Total protein	-0.340	-0.78	0.10	0.13	148	1224	2	71.44	3.50	0.07
Chlorine	-0.179	-0.42	0.06	0.14	75	1021	2	0.00	0.00	0.00
Fibrinogen	0.671	-0.21	1.55	0.14	148	1224	2	92.02	12.53	0.37
Platelet-to-WBC ratio	-1.912	-4.51	0.68	0.15	257	586	2	99.49	196.55	3.49
Lactate dehydrogenase	0.290	-0.12	0.70	0.17	73	823	2	59.47	2.47	0.05
Albumin-to-globulin ratio	-0.511	-1.26	0.24	0.18	82	1167	2	87.49	7.99	0.26
Monocyte count	0.134	-0.07	0.34	0.20	405	1939	5	65.29	11.52	0.03
GOT/AST	0.482	-0.28	1.24	0.21	130	1867	3	94.28	34.95	0.43
Procalcitonin (PCT)	0.295	-0.19	0.78	0.24	139	1108	4	83.14	17.80	0.20
Glucose	0.232	-0.16	0.62	0.24	230	2401	3	86.53	14.84	0.10
Interleukin (IL)-6	0.389	-0.30	1.08	0.27	142	969	4	91.53	35.44	0.45
Bilirubin	0.163	-0.13	0.46	0.28	97	1607	2	51.87	2.08	0.02
Prothrombin time	0.251	-0.24	0.75	0.32	148	1224	2	77.30	4.41	0.10
Neutrophil-to-lymphocyte ratio	0.190	-0.20	0.58	0.34	810	3866	9	95.71	186.32	0.34
Potassium	-0.214	-0.66	0.23	0.34	223	2245	4	87.74	24.47	0.18
Haematocrit	0.086	-0.12	0.29	0.42	93	1866	2	0.00	0.81	0.00
Creatinine	-0.109	-0.46	0.24	0.54	419	4928	7	91.09	67.34	0.20
Cholesterol	-0.096	-0.43	0.23	0.57	168	2267	2	73.66	3.80	0.04
Lymphocyte-to-monocyte ratio	-0.063	-0.28	0.15	0.57	115	300	2	0.00	0.47	0.00
Triglyceride	-0.046	-0.20	0.11	0.57	168	2267	2	0.00	0.15	0.00
Activated partial thromboplastin time	-0.048	-0.22	0.13	0.59	148	1224	2	0.00	0.90	0.00
Uric acid	0.117	-0.33	0.56	0.60	148	1224	2	72.37	3.62	0.08
Alanine transaminase	-0.063	-0.37	0.24	0.69	236	2330	4	76.21	12.61	0.07
Partial pressure of carbon dioxide in arterial blood (PaC02)	0.040	-0.19	0.27	0.74	88	1106	2	9.47	1.10	0.00
Neutrophil count	-0.072	-0.51	0.37	0.75	645	4320	7	95.88	145.70	0.33
Aspartate transaminase	-0.019	-0.19	0.15	0.83	148	1224	2	0.00	0.45	0.00
High density lipoprotein	-0.015	-0.17	0.14	0.85	168	2267	2	0.00	0.02	0.00
Partial pressure of oxygen in arterial blood (Pa02)	-0.089	-1.01	0.83	0.85	88	1106	2	93.86	16.30	0.41
White blood cell count (WBC)	0.037	-0.41	0.48	0.87	803	6270	13	96.69	362.63	0.63
Thrombin time	-0.011	-0.18	0.16	0.90	148	1224	2	0.00	0.85	0.00
Platelet count	0.004	-0.07	0.08	0.91	929	6989	13	0.00	7.15	0.00
Urea	-0.046	-2.41	2.32	0.97	73	823	2	98.60	71.34	2.88

SMD and 95% CI of the random effects model meta-analysis between POP and no-POP for pre-operative biomarkers in 2 or more studies. Biomarkers in bold = statistically significant biomarkers (*p* value ≤ 0.05)

*Abbreviations*: *SMD *standardised mean difference, *LL *lower limit, *POP *post-operative pneumonia, *UL *upper limit

**Table 3:** Post-operative biomarkers reported in$\:\ge\:$2 studies

Biomarker	SMD	Lower Limit	Upper Limit	*p*	Sample Size		k	I^2^	Q	Tau^2^
					POP	No-POP				
Day 1 Post-Operative Biomarkers										
PCT	0.684	0.05	1.31	**0.03**	514	2875	12	97.25	399.81	1.17
IL-6	0.640	-0.11	1.39	0.09	109	910	3	90.26	20.54	0.39
WBC	0.216	-0.13	0.56	0.22	340	2368	8	87.70	56.93	0.22
CRP	-0.090	-0.57	0.39	0.72	295	2115	7	92.36	78.52	0.39
Creatinine	0.055	-0.31	0.42	0.77	105	934	2	68.39	3.16	0.05
High sensitivity CRP	0.000	-0.21	0.21	1.00	107	522	2	0.00	0.00	0.00
Day 2 Post-Operative Biomarkers										
PCT	1.344	0.65	2.04	**0.0001**	318	1685	7	95.81	143.11	0.79
CRP	0.264	0.03	0.49	**0.02**	252	1505	5	57.88	9.50	0.04
IL-6	-0.708	-1.73	0.32	0.18	92	874	2	94.22	17.29	0.51
WBC	0.398	-0.23	1.02	0.21	212	1326	5	93.24	59.14	0.47
Day 3 Post-Operative Biomarkers										
PCT	1.165	0.52	1.81	**0.0004**	335	1823	8	95.41	152.51	0.77
IL-6	0.723	0.28	1.16	**0.001**	92	874	2	69.39	3.27	0.07
WBC	1.303	0.29	2.32	**0.01**	212	1326	5	97.07	136.43	1.25
CRP	0.425	-0.07	0.92	0.09	269	1643	6	91.52	58.93	0.34
Day 4 Post-Operative Biomarkers										
CRP	0.679	0.53	0.83	**< 0.0001**	212	1465	4	0.00	2.26	0.00
IL-6	0.751	0.36	1.15	**0.0002**	92	874	2	61.83	2.62	0.05
WBC	0.285	0.12	0.45	**0.001**	172	1286	4	0.00	1.67	0.00
PCT	0.379	-0.14	0.90	0.15	278	1645	6	92.65	68.04	0.37
Day 5 Post-Operative Biomarkers										
CRP	1.158	0.82	1.50	**< 0.0001**	212	1465	4	77.48	13.32	0.09
PCT	0.357	-0.08	0.79	0.11	278	1645	6	89.58	47.99	0.25
WBC	-0.151	-0.56	0.26	0.47	172	1286	4	82.84	17.48	0.14
IL-6	0.052	-0.64	0.74	0.88	109	910	3	88.68	17.67	0.33
Day 6 Post-Operative Biomarkers										
PCT	1.001	0.77	1.23	**< 0.0001**	106.000	359.000	2	0.00	0.81	0.00

SMD and 95% CI of the random effects model meta-analysis between POP and no-POP for post-operative biomarkers reported in 2 or more studies by timepoint. Biomarkers in bold = statistically significant biomarkers (*p* value ≤ 0.05)

*Abbreviations*: *CRP *C-Reactive Protein, *IL-6 *interleukin-6, *PCT *procalcitonin, *POP *post-operative pneumonia, *SMD *standardised mean difference, *WBC *white blood cell count

#### Meta-analysis of pre-operative biomarkers

**Fig. 4:**
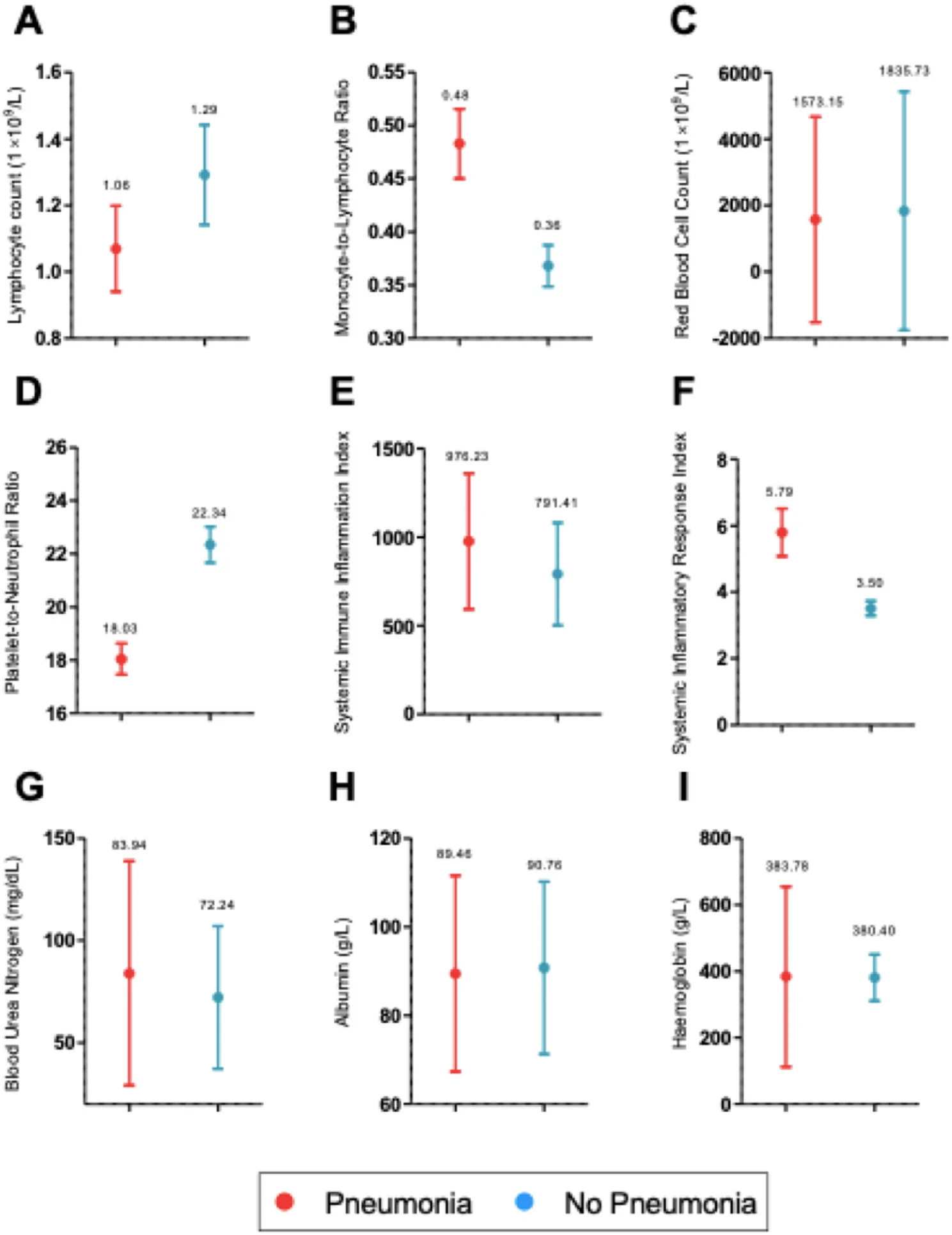
Pre-operative raw mean values for statistically significant biomarkers. Data is presented in weighted mean and 95% confidence interval for pneumonia and no-pneumonia. **A** Lymphocyte count (x10^9^/L)*. **B** Monocyte-to-lymphocyte ratio. **C** Red blood cell count (x10^9^/L). **D** Platelet-to-neutrophil ratio. **E** Systemic immune inflammation index. **F** Systemic inflammatory response index. **G** Blood urea nitrogen (mg/dL). **H** Albumin (g/L)*. **I** Haemoglobin (g/L)*. Note: units of measure were standardised where possible, * indicates that studies were excluded where unit of measure was not provided

Patients who developed pneumonia after surgery demonstrated significantly lower platelet-to-neutrophil ratio (SMD - 0.55 [CI -0.69; -0.40], *p* < 0.0001), red blood cell count (SMD -0.44 [CI -0.71; -0.17], *p* = 0.001), haemoglobin (SMD -0.24 [CI -0.39; -0.09], *p* = 0.002), albumin (SMD -0.46 [CI -0.77; -0.14], *p* = 0.005) and lymphocyte count (SMD -0.24 [CI -0.42; -0.06], *p* = 0.008) at pre-operative baseline compared to those who did not develop pneumonia. Significantly higher monocyte-to-lymphocyte ratio (SMD 0.51 [CI 0.36–0.66], *p* < 0.0001), systemic immune inflammation index (SII) (calculated by neutrophil count × platelet count / lymphocyte count [29]) (SMD 0.66 [CI 0.37–0.95], *p* < 0.0001), systemic inflammatory response index (SIRI) (calculated by neutrophil count × monocyte count / lymphocyte count [30]) (SMD 0.64 [CI 0.39–0.89], *p* < 0.0001) and blood urea nitrogen (SMD 0.43 [CI 0.21–0.64], *p* = 0.0001) were also observed pre-operatively (Table 2). No significant difference was observed for the 39 remaining pre-operative biomarkers. The mean values of the significant pre-operative biomarkers are presented in Fig. 4.

Although pooled estimates were statistically significant, the results should be interpreted with caution given the considerable heterogeneity (Additional File 6). Albumin (I^2^ = 91.4%), lymphocyte count (I^2^ = 79.9%) and SII (I^2^ = 77.2%) demonstrated considerable heterogeneity, and SIRI (I^2^ = 64.6%), haemoglobin (I^2^ = 61.7%) and blood urea nitrogen (I^2^ = 56.1%) demonstrated substantial heterogeneity. Low heterogeneity was observed for platelet-to-neutrophil ratio (I^2^ = 0.0%) and monocyte-to-lymphocyte ratio (I^2^ = 0.0%), and low-moderate heterogeneity for red blood cell count (I^2^ = 35.3%), but meta-analyses on these three biomarkers were based on low study numbers (k = 2).

Due to limited study numbers, Egger’s test was only feasible pre-operatively for haemoglobin, and did not demonstrate statistically significant funnel plot asymmetry (*p* = 0.22), suggesting no clear evidence of small-study effects. Sensitivity analysis excluding studies at high or very high risk of bias was also only feasible for haemoglobin, leaving four studies all classified as having some concerns of bias (Additional File 6). Although the direction and pooled effect size were largely unchanged, the association was no longer statistically significant (SMD − 0.20 [CI -0.58–0.17], *p* = 0.29), with considerable heterogeneity persisting (I^2^ = 83.0%) (Additional File 6). Similar findings were observed after excluding haemoglobin studies that required data conversion (SMD − 0.18 [CI -0.38–0.01], *p* = 0.07), although heterogeneity was reduced (I^2^ = 39.0%) (Additional File 6). When removing studies with data conversion for albumin and lymphocyte count the pooled effect estimate and statistical significance remained largely unchanged, yet heterogeneity reduced, particularly for lymphocyte count (SMD − 0.49 [CI -0.82 – -0.15], *p* = 0.004, I^2^ = 79.0%; and SMD − 0.34 [CI -0.46 – -0.21], *p* < 0.00001, I^2^ = 0.0%), respectively (Additional File 6).

#### Meta-analysis of post-operative biomarkers

Patients who developed pneumonia after surgery demonstrated higher procalcitonin (PCT) at day one post-operatively (SMD 0.68 [CI 0.05–1.31], *p* = 0.03), day two (SMD 1.34 [CI 0.65–2.04], *p* = 0.0001), day three (SMD 1.17 [CI 0.52–1.81], *p* = 0.0004) and day six (SMD 1.00 [CI 0.77–1.23], *p* < 0.0001); higher C-Reactive Protein (CRP) at day two (SMD 0.26 [CI 0.03–0.49], *p* = 0.02), day four (SMD 0.68 [CI 0.53–0.83], *p* < 0.0001) and day five (SMD 1.16 [CI 0.82–1.50], *p* < 0.0001); higher interleukin (IL)-6 at day three (SMD 0.72 [CI 0.28–1.16], *p* = 0.001) and day four (SMD 0.75 [CI 0.36–1.15], *p* = 0.0002); and higher white blood cell (WBC) count at day three (SMD 1.30 [CI 0.29–2.32], *p* = 0.01) and day four (SMD 0.29 [CI 0.12–0.45], *p* = 0.001), than those without POP (Table 3).

**Fig. 5:**
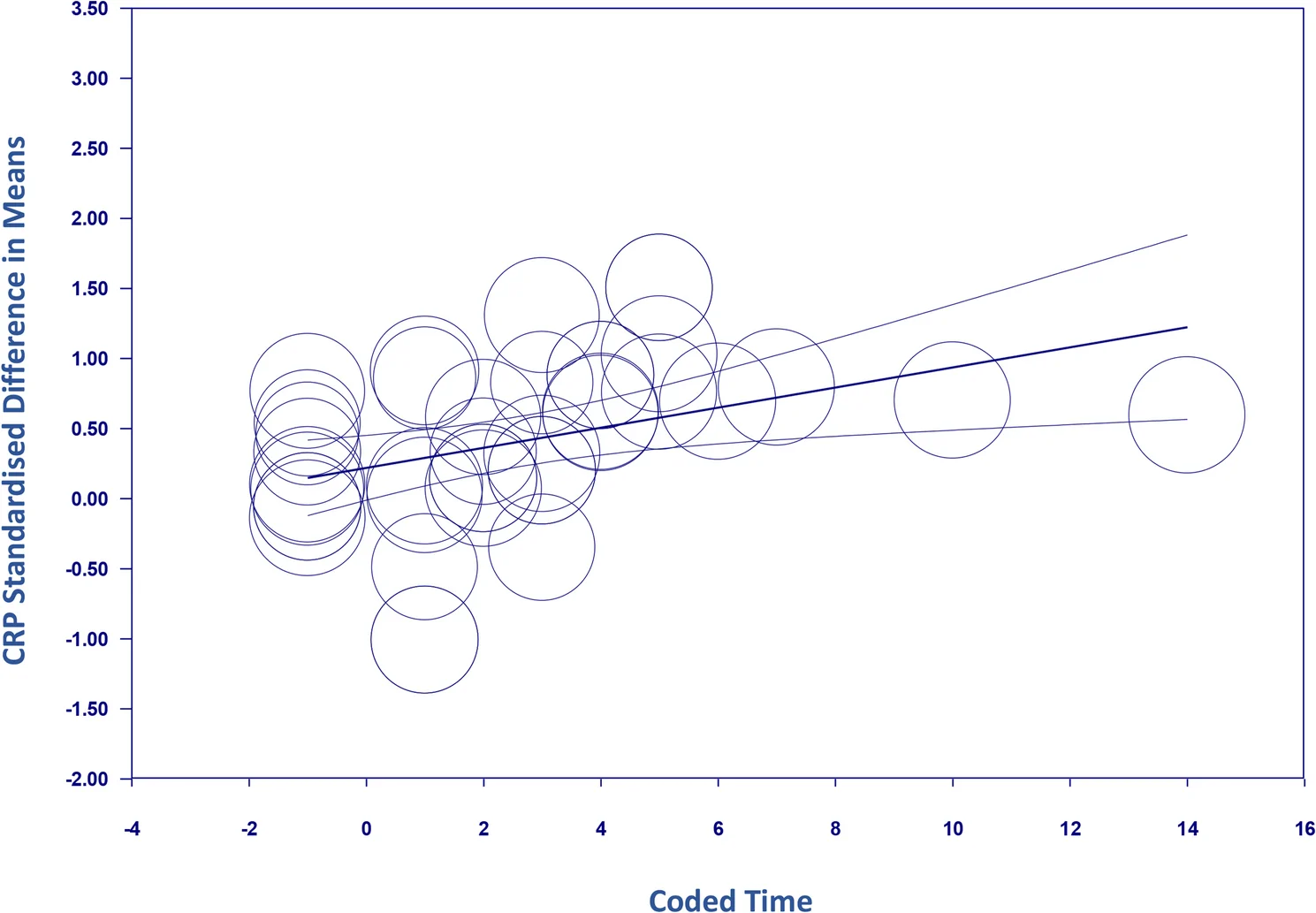
Meta-regression analysis of changes in C-reactive protein over time. Meta-regression analysis of SMD between pneumonia and no-pneumonia for C-Reactive Protein with pre-operative and post-operative assessments. Key for coded time: -1 = pre-operative baseline; 1–10 = day post-operatively. Each circle represents one study

There was a positive correlation between the SMD for CRP and time, demonstrating that there was a greater difference in CRP between groups (higher for pneumonia) as the number of days passed after surgery (*p* = 0.002, R^2^ = 0.18) (Fig. 5). Results for IL-6 (*p* = 0.85), PCT (*p* = 0.71) and WBC (*p* = 0.40) were not statistically significant.

Several pooled analyses of post-operative biomarkers demonstrated considerable heterogeneity (I² >75%); therefore, statistically significant pooled estimates were interpreted as exploratory and were further evaluated using sensitivity analyses. Sensitivity analysis, excluding studies at high or very high risk of bias, was only possible for PCT, CRP and WBC, at some of the post-operative timepoints, due to low study numbers (Additional File 6). Sensitivity analysis by exclusion of studies requiring conversion of summary statistics was also not possible.

For CRP, the pooled effect size, direction and heterogeneity were largely unchanged following sensitivity analysis for day two (SMD 0.12 [CI -0.42–0.65], *p* = 0.66, I^2^ = 85.0%), day four (SMD 0.72 [CI 0.53–0.91], *p* < 0.00001, I^2^ = 0.0%) and day five (SMD 1.23 [CI 0.68–1.78], *p* < 0.0001, I^2^ = 85.0%) post-operatively, yet results were no longer statistically significant at day two and were reported in low study numbers (k = 3) (Additional File 6). For WBC, the pooled effect size, direction and heterogeneity were also largely unchanged at day four, with results remaining statistically significant (SMD 0.26 [CI 0.07–0.45], *p* = 0.009, I^2^ = 0.0%). At day three, sensitivity analysis substantially reduced the pooled effect size for WBC, and reduced heterogeneity (SMD − 0.13 [CI -0.32–0.06], *p* = 0.18, I^2^ = 0.0%), yet the association was no longer statistically significant (Additional File 6).

For PCT, sensitivity analysis substantially reduced the pooled effect size at all available timepoints, yet remained statistically significant (day one: SMD 0.95 [CI 0.47–1.42], *p* = 0.0001; day two: SMD 0.81 [CI 0.25–1.37], *p* = 0.004; and day three SMD 0.74 [CI 0.56–0.92], *p* < 0.00001) (Additional File 6). Substantial heterogeneity remained at day one (I^2^ = 89.0%) and day two (I^2^ = 86.0%), yet was reduced at day three (I^2^ = 0.0%) post-operatively. Egger’s test was only feasible for PCT at day one, and did not demonstrate statistically significant funnel plot asymmetry (*p* = 0.55).

### Association between pneumonia and prognostic outcomes

**Fig. 6:**
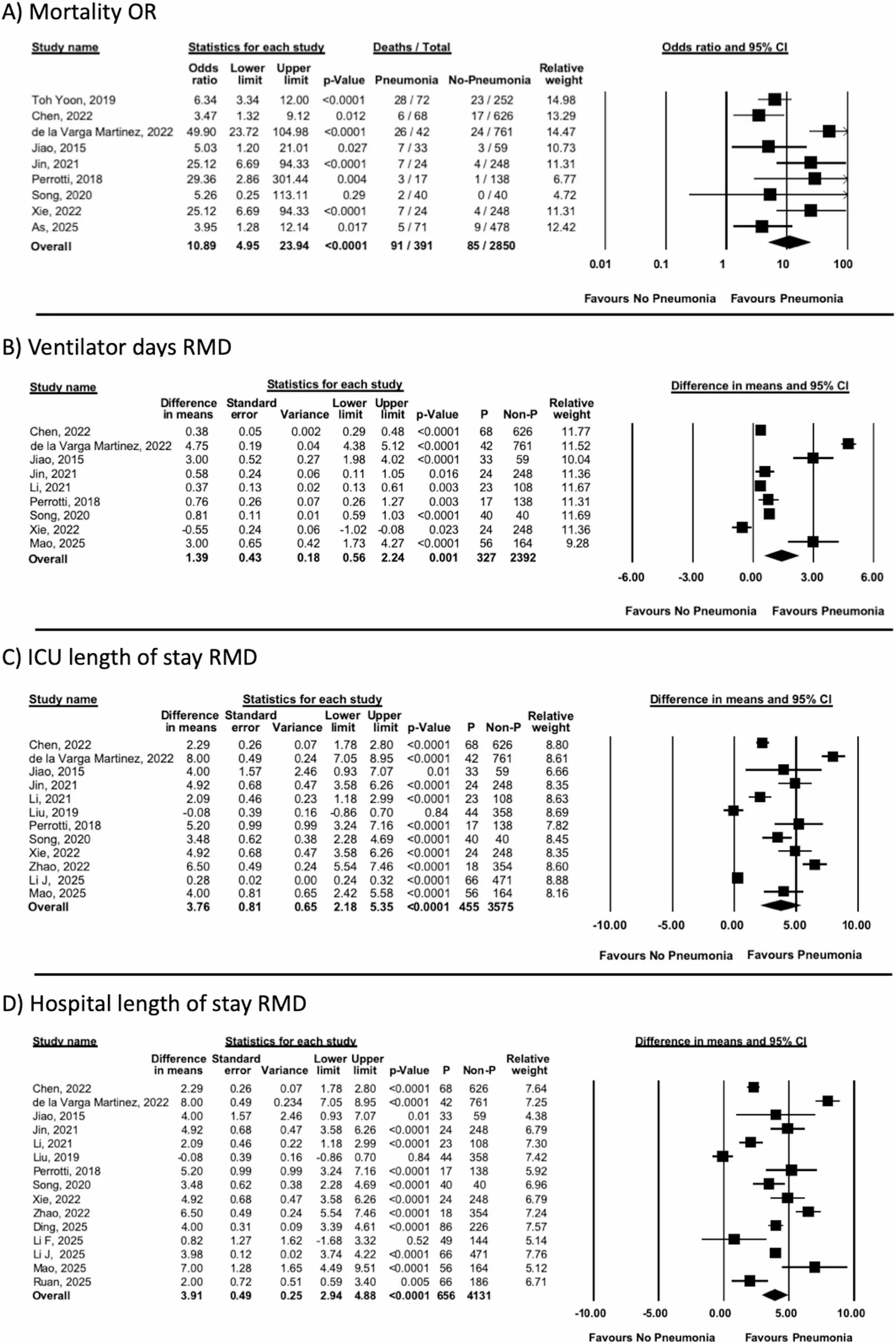
Forest plots for prognostic outcome data in pneumonia and no-pneumonia groups. **A** Mortality OR; (**B**) Ventilator days RMD; (**C**) ICU length of stay RMD; (**D**) Hospital length of stay RMD. OR: odds ratio; RMD: raw mean difference; ICU: intensive care unit; CI: confidence interval; P: pneumonia group; Non-P: no-pneumonia group. Note: Ozawa [44] excluded from mortality analysis as zero events reported in both groups and not possible for calculation on the meta-analysis software. Li, J [41] excluded from ventilator days analysis as uncertain reporting of measure of variance and therefore missing mean and standard deviation data

**Fig. 7:**
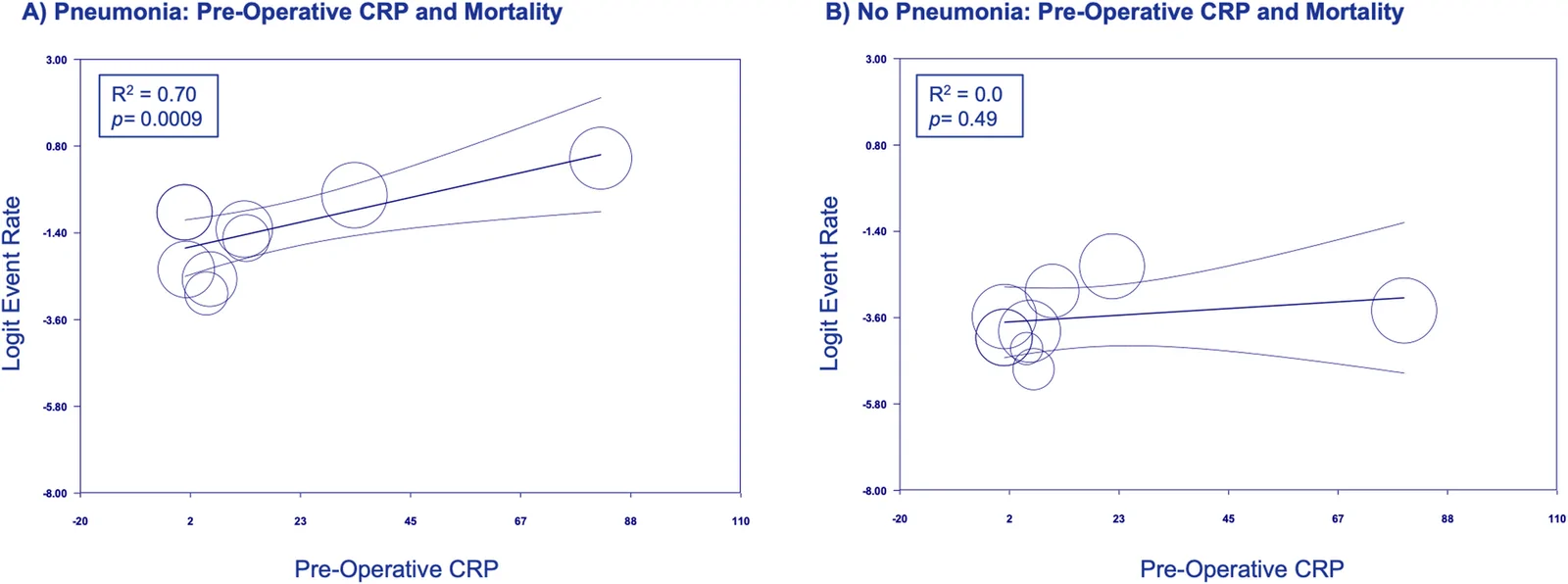
Meta-regression for mortality and pre-operative C-reactive protein. Meta-regression analysis of pre-operative C-Reactive Protein (CRP) raw values and mortality in (**A**) pneumonia group, and (**B**) no-pneumonia group. Each circle represents one study and the size of the circle represents the weight of the study

Patients who developed POP had significantly higher mortality rates (*p* < 0.0001, Fig. 6A), ventilator days (*p* = 0.001, Fig. 6B), ICU length of stay (*p* < 0.0001, Fig. 6C) and hospital length of stay (*p* < 0.0001, Fig. 6D) compared to those without pneumonia. Meta-regression analysis identified a statistically significant correlation between pre-operative CRP and mortality in patients who developed POP (*p* = 0.0009, Fig. 7A), not evident in those without pneumonia (0.49, Fig. 7B). All other prognostic outcome variables were not feasible for testing in association with biomarker data, as they were reported in low study numbers.

## Discussion

This study synthesised the evidence for blood-based biomarkers of POP. Thirty-seven studies consisting of 15,842 surgical patients were included, with an overall pneumonia rate of 17.8%. Pre-operatively, patients who developed POP showed higher monocyte-to-lymphocyte ratio, SIRI, SII, and blood urea nitrogen, and lower platelet-to-neutrophil ratio, red blood cell count, haemoglobin, albumin, and lymphocyte count, suggesting early associative biomarker signals. Post-operatively, POP was associated with higher CRP, IL-6, PCT, and WBC within the first six days after surgery. However, interpretation is limited by heterogeneous pneumonia definitions and inconsistent reporting of diagnosis timing across studies, in addition to high risk of bias. Nonetheless, patients with POP also experienced worse outcomes, including increased mortality, longer ventilation, and prolonged ICU and hospital stay, confirming the importance of an improved understanding of POP.

The nine statistically significant pre-operative biomarkers broadly reflect nutritional status, systemic inflammation, and physiological reserve, all of which are known to influence surgical outcomes [50]. Lower albumin and lymphocyte counts, alongside elevated inflammatory indices, may indicate increased baseline vulnerability [50, 51]. Several biomarkers, including platelet-neutrophil ratio, monocyte-lymphocyte ratio, monocytes, blood urea nitrogen and SIRI, have also been associated with malnutrition, frailty and systemic inflammation [52–55]. However, these findings were derived from a limited number of heterogeneous studies, and may reflect general susceptibility to post-operative complications rather than pneumonia-specific pathology. These associative biomarker signals should therefore be considered exploratory and hypothesis-generating, despite their statistical significance.

Post-operative biomarkers, including CRP, IL-6, WBC and PCT, were consistently associated with POP within the first six-days following surgery. However, these biomarkers are non-specific indicators of inflammation and may be influenced by surgical trauma, illness severity, and other peri-operative complications including anastomotic leak and delirium [56–58]. Interpretation is further limited by the potential for diagnostic bias, as these routinely available biomarkers may have informed POP diagnosis in unblinded clinical settings and observational study designs. Nevertheless, given their widespread clinical availability, these associative biomarker signals may benefit from further rigorous evaluation and the development of POP-specific clinical thresholds.

Overall, although several biomarkers differed significantly between patients with and without post‑operative pneumonia, the observed associations may represent non-specific inflammatory responses rather than clinically actionable diagnostic tools. Most studies reported group-level differences without diagnostic thresholds or performance metrics, precluding pooled accuracy analyses and preventing identification of clinically meaningful cut-offs. Future research should prioritise prospective designs, standardised POP definitions, pre-specified biomarker thresholds, and formal diagnostic accuracy metrics. Collectively, these findings should be regarded as associative and hypothesis‑generating, rather than evidence of diagnostic or prognostic utility.

Furthermore, the substantial clinical heterogeneity across studies, including surgical type, patient characteristics such as age or premorbid status, and peri-operative context, limits the generalisability of findings. Biomarker responses may therefore reflect diverse biological processes across critically ill populations. However, these factors were not possible to evaluate as part of analysis due to inconsistencies in study reporting. In addition, variability in pneumonia definitions and diagnostic criteria introduces risk of outcome misclassification and further constrains interpretation. Although this is reflective of the challenges faced in clinical practice and previously published studies, the lack of consistency in pneumonia diagnosis impedes the results, due to variations in biomarker reference standards [59]. Diagnostic discrepancies may also occur due to the geographical variation of the included studies. The predominance of studies conducted in China also limits global applicability, due to potential variation in patient demographics, surgical practices, and peri-operative care, including prophylactic antibiotic use and POP diagnostics.

### Strength and certainty of evidence

The certainty of evidence for all identified biomarker associations was judged to be low to very low, based on several consistent limitations across the evidence base:

#### Risk of bias

All included studies were observational and more than one third were rated at very high overall risk of bias, primarily due to confounding and post‑exposure interventions. Sensitivity analyses excluding high and very high‑risk studies demonstrated attenuation of several pooled estimates, highlighting limited robustness.

#### Inconsistency

Statistical heterogeneity was substantial-to-extreme for many pooled analyses (often I² >75–90%), indicating considerable variability in effect estimates across studies. In such settings, statistically significant pooled effects should be interpreted cautiously, as they may not represent a single underlying association.

#### Indirectness

The included studies encompassed heterogeneous surgical populations, peri‑operative contexts, pneumonia subtypes, and diagnostic criteria. This clinical and methodological variability limits the direct applicability of pooled estimates to any specific surgical setting or pneumonia phenotype.

#### Imprecision

Many pooled analyses were based on small numbers of studies, with wide confidence intervals and limited power to support stable inference.

### Limitations

This review has several notable strengths, including its large pooled sample size, extensive biomarker synthesis, and its novelty as the first review, to our knowledge, to comprehensively evaluate biomarkers associated with POP across surgical populations. Nevertheless, several limitations should also be acknowledged. Restriction to English-language, full-text studies and exclusion of grey literature may have introduced publication bias. The search strategy limitations (title and abstract) and single-reviewer data extraction (with verification) may have increased risk of missed studies and extraction errors. While broad search terms were used to maximise sensitivity, some relevant studies may nevertheless have been missed. The external validity is also limited as seventeen of the included studies are from China, thus limiting their application to wider clinical practice and impeding global generalisability.

Clinical heterogeneity, high risk of bias and low study numbers further limit robustness. In particular, the heterogeneous diagnostic criteria and limited reporting of diagnosis timing introduces a high risk of outcome misclassification. This limitation substantially constrains interpretation and precludes diagnostic or mechanistic inference. Additionally, conversion of non-parametric data (median/IQR) to mean/SD may have also introduced bias. Although commonly applied in meta‑analyses, this approach assumes approximately symmetric distributions and may introduce distortion when biomarkers are highly skewed, as is plausible for inflammatory markers such as CRP, IL‑6, and PCT. Consequently, some pooled estimates may be influenced by distributional assumptions rather than true differences between groups. Furthermore, given the substantial burden of multiple comparisons across 115 biomarkers and numerous timepoints, some of our statistically significant findings may be chance findings. Whilst our findings remain exploratory, the results require future validation. Where feasible, sensitivity analyses were conducted to assess robustness; however, the limited number of studies constrained these analyses. This limitation further supports cautious interpretation of pooled effect estimates.

### Future directions

Future studies should adopt prospective designs with standardised POP definitions and consistent biomarker sampling protocols. Robust strategies to minimise confounding, such as comprehensive measurement of baseline characteristics and appropriate multivariable adjustment are also essential. In addition, the impact of post-exposure interventions, including variations in peri-operative care and antibiotic use, should be explicitly accounted for through study design and reporting. Establishing validated, clinically relevant biomarker thresholds and conducting external validation in diverse populations will be essential for improving reliability and enabling clinical application. Finally, external validation in independent and geographically diverse cohorts will be critical to strengthen the reliability and global generalisability of findings.

## Conclusions

Our study highlights pre-operative and post-operative blood-based biomarker signals associated with POP in observational studies. Monocyte-to-lymphocyte ratio, SIRI, SII, and blood urea nitrogen, platelet-to-neutrophil ratio, red blood cell count, haemoglobin, albumin, and lymphocyte count were identified as associative biomarker signals for pneumonia prior to surgery. Within the first six days after surgery, CRP, WBC, IL-6 and PCT were identified as post-operative biomarker signals. Furthermore, pre-operative CRP was associated with increased mortality within the patients who later developed POP. However, the high risk of bias and heterogeneity across pneumonia definitions, clinical reference standards, biomarker testing methods and surgical populations, means that these biomarkers cannot currently be used for prediction or diagnosis in clinical practice. Given low certainty of evidence due to risk of bias, heterogeneity, indirectness, and imprecision, these findings should be interpreted as hypothesis‑generating rather than clinically determinative.

Taken together, this review identifies associative biomarker signals observed across heterogeneous surgical and clinical contexts. Future research should prioritise prospective studies with standardised diagnostic definitions of POP, improved control of confounding, and the development and external validation of clinically meaningful biomarker thresholds to improve the validity, reproducibility, and clinical applicability of biomarker research. Our study reinforces the need for a standardised approach to pneumonia outcome collection, not only to improve the validity and utility of future research, but to support future development of diagnostic testing and targeted prevention and treatment therapies. The results confirm the deleterious consequences of POP, warranting further research to improve patient outcomes after surgery.

## Supplementary Information

Additional file 1.

Additional file 2.

Additional file 3.

Additional file 4.

Additional file 5.

Additional file 6.

## Data Availability

The datasets supporting the conclusions of this article are included within the article and its additional files.
