# Supplementary material for: Biomarkers associated with post-operative pneumonia: a systematic review and meta-analysis
Source: BMC Pulm Med. 2026 Jul 16;26:455. doi: 10.1186/s12890-026-04486-7 (PMC13651040; doi:10.1186/s12890-026-04486-7)
Supplement: Supplementary file 2 [file 12890_2026_4486_MOESM2_ESM.docx]

**Additional File 2**

**Syntax:**

Preliminary scoping searches identified predominantly observational cohort studies. The PECO syntax was therefore used to determine key words and inclusion criteria;

- Population: humans, adults (aged $\geq$18 years), hospitalised after any type of surgery
- Exposure – diagnosed with post-operative pneumonia after any type of surgery
- Control – no post-operative pneumonia
- Outcome – any blood-based biomarker

**Keywords, synonyms, heading (MeSH) and entry terms for systematic search strategy:**

| Keyword | Entry terms search within Abstract and Title |
| --- | --- |
| Biomarkers | ‘biomarker*‘ OR ‘Marker* Biologic*‘ OR ‘Biologic* Marker*’ OR ‘Marker* Immun*’ OR ‘Immun* Marker*’ OR ‘Serum Marker*’ OR ‘Marker* Serum’ OR ‘Surrogate End point*’ OR ‘Endpoint* Surrogate*’ OR ‘Surrogate endpoint*’ OR ‘End Point*, Surrogate’ OR ‘Marker* Clinical’ OR ‘Clinical Marker*’ OR ‘Viral Marker*’ OR ‘Marker* Viral’ OR ‘biochemical marker*’ OR ‘marker biochemical*’ OR ‘laboratory marker*’ OR ‘marker* laboratory’ OR ‘surrogate marker*’ OR ‘marker* surrogate’ OR ‘blood marker*’ OR ‘marker* blood’ |
| Post-Operative Pneumonia | ‘Pneumonia*’ OR ‘pneumonitis’ OR ‘Pneumonitides’ OR ‘Pulmonary Inflammation’ OR ‘Inflammation, Pulmonary’ OR ’Lung Inflammation’ OR ‘Inflammation, Lung’ OR ‘Respiratory tract infection’ OR ‘Bronchopneumonia’ OR ‘Lobar Pneumonia’ OR ‘Post-Operative Complication*’ OR ‘Postoperative complication’ OR ‘Complication*, Post-Operative’ OR ‘Complication*, Postoperative’ OR ‘infective complication*’ OR ‘complication*, infective’ OR ‘pulmonary complication*’ OR ‘complication* pulmonary’ OR ‘postoperative pulmonary complication*’ OR ‘complication*, postoperative pulmonary’ OR ‘post-operative pulmonary complication*’ OR ‘complication*, post-operative pulmonary’ OR ‘PPC’ |
| Surgery | ‘Surgical Procedure*, Operative’ OR ‘Surgical Procedure’ OR ‘Procedure*, Surgical’ OR ‘Operative Procedure*’ OR ‘Procedure*, Operative’ OR ‘Operative Surgical Procedure*’ OR ‘Procedure*, Operative Surgical’ OR ‘Surgical Procedure*, Operative’ OR ‘General Surgery’ OR ‘Surgery, General’ OR ‘Surgery’ OR ‘Surgical’ OR ‘Surg’ OR ‘Operation’ OR ‘invasive procedure*’ OR ‘operative therap*’ OR ‘operative procedure*’ OR ‘procedure* *operative’ OR ‘procedure’ OR ‘transplant*’ OR ‘*section’ OR ‘*otomy’ OR ‘*ectomy’ OR ‘*scopy’ OR ‘*plasty’ OR ‘replacement’ OR ‘debridement’ OR ‘graft’ OR ‘repair’ OR ‘reconstruction’ OR ‘fusion’ OR ‘fixation’ OR ‘reduction’ OR ‘release’ OR ‘decompression’ |

**PubMed Search Strategy**

("biomarkers"[mh] OR "biomarker*"[tiab] OR “Marker*, Biologic*”[tiab] OR “Biologic* Marker*”[tiab] OR “Marker*, Immunologic*”[tiab] OR “Immune Marker*”[tiab] OR “Marker*, Immune”[tiab] OR “Immunologic* Marker*”[tiab] OR “Serum Marker*”[tiab] OR “Marker*, Serum”[tiab] OR “Surrogate Endpoint*”[tiab] OR “Endpoint*, Surrogate”[tiab] OR “Surrogate End Point*”[tiab] OR “End Point*, Surrogate”[tiab] OR “Marker*, Clinical”[tiab] OR “Clinical Marker*”[tiab] OR “Viral Marker*”[tiab] OR “Marker*, Viral”[tiab] OR “Biochemical Marker*”[tiab] OR “Marker*, Biochemical”[tiab] OR “Marker*, Laboratory”[tiab] OR “Laboratory Marker*”[tiab] OR “Blood Marker*”[tiab] OR “Marker*, Blood”[tiab])

**AND**

(“Pneumonia”[mh] OR “Pneumonia"[tiab] OR “pneumonias”[tiab] OR “pneumonitis”[tiab] “Pneumonitides”[tiab] OR “Pulmonary Inflammation”[tiab] OR “Inflammation, Pulmonary”[tiab] OR “Lung Inflammation”[tiab] OR “Inflammation, Lung”[tiab] OR “Respiratory tract infection”[tiab] OR “Bronchopneumonia”[tiab] OR “Lobar Pneumonia”[tiab] OR “Post-Operative Complication"[mh] OR “Post-Operative Complication*"[tiab] OR “Complication*, Post-Operative*"[tiab] OR “Complication*, Postoperative"[tiab] OR “Postoperative Complication*"[tiab] OR “Infective Complication*"[tiab] OR “Complication*, Infective"[tiab] OR “Pulmonary Complication*"[tiab] OR “Complication* Pulmonary"[tiab] OR “postoperative pulmonary complication*"[tiab] OR “pulmonary complication*"[tiab] OR “complication*, pulmonary"[tiab] OR “complication*, postoperative pulmonary"[tiab] OR “post-operative pulmonary complication*"[tiab] OR “complication*, post-operative pulmonary"[tiab] OR “PPC”[tiab])

**AND**

(“Surgical Procedure, Operative”[mh] OR “Surgical Procedure*, Operative"[tiab] OR “Surgical Procedure*"[tiab] OR “Procedure*, Surgical"[tiab] OR “Operative Procedure*"[tiab] OR “Procedure*, Operative"[tiab] OR “Operative Surgical Procedure*"[tiab] OR “Procedure*, Operative Surgical"[tiab] OR “Surgical Procedure*, Operative"[tiab] OR “General Surgery”[mh] OR “General Surgery"[tiab] OR “Surgery, General"[tiab] OR “Surgery”[mh] OR “Surgery"[tiab] OR “Surgical"[tiab] OR “Surg"[tiab] OR “Operation*"[tiab] OR “invasive procedure*"[tiab] OR “operative therap*”[tiab] OR “operative procedure*"[tiab] OR “procedure* *operative"[tiab] OR “procedure"[tiab] OR “transplant*"[tiab] OR “section"[tiab] OR “otomy"[tiab] OR “ectomy"[tiab] OR “scopy"[tiab] OR “plasty"[tiab] OR “replacement"[tiab] OR “debridement"[tiab] OR “graft*"[tiab] OR “repair"[tiab] OR “reconstruction"[tiab] OR “fusion"[tiab] OR “fixation"[tiab] OR “reduction"[tiab] OR “release"[tiab] OR “decompression"[tiab])

**Cochrane Search Strategy**

(‘biomarker*‘ OR ‘Marker* Biologic*‘ OR ‘Biologic* Marker*’ OR ‘Marker* Immun*’ OR ‘Immun* Marker*’ OR ‘Serum Marker*’ OR ‘Marker* Serum’ OR ‘Surrogate End point*’ OR ‘Endpoint* Surrogate*’ OR ‘Surrogate endpoint*’ OR ‘End Point*, Surrogate’ OR ‘Marker* Clinical’ OR ‘Clinical Marker*’ OR ‘Viral Marker*’ OR ‘Marker* Viral’ OR ‘biochemical marker*’ OR ‘marker biochemical*’ OR ‘laboratory marker*’ OR ‘marker* laboratory’ OR ‘surrogate marker*’ OR ‘marker* surrogate’ OR ‘blood marker*’ OR ‘marker* blood’ **ti,ab,kw**

**OR**

MeSH term: *“Biomarker”*

**AND**

(‘Pneumonia*’ OR ‘pneumonitis’ OR ‘Pneumonitides’ OR ‘Pulmonary Inflammation’ OR ‘Inflammation, Pulmonary’ OR ’Lung Inflammation’ OR ‘Inflammation, Lung’ OR ‘Respiratory tract infection’ OR ‘Bronchopneumonia’ OR ‘Lobar Pneumonia’ OR ‘Post-Operative Complication*’ OR ‘Postoperative complication’ OR ‘Complication*, Post-Operative’ OR ‘Complication*, Postoperative’ OR ‘infective complication*’ OR ‘complication*, infective’ OR ‘pulmonary complication*’ OR ‘complication* pulmonary’ OR ‘postoperative pulmonary complication*’ OR ‘complication*, postoperative pulmonary’ OR ‘post-operative pulmonary complication*’ OR ‘complication*, post-operative pulmonary’ OR ‘PPC’)**ti,ab,kw**

**OR**

MeSH term: *“Pneumonia”*

**OR**

MeSH term: *‘Postoperative complication”*

**AND**

(‘Surgical Procedure*, Operative’ OR ‘Surgical Procedure’ OR ‘Procedure*, Surgical’ OR ‘Operative Procedure*’ OR ‘Procedure*, Operative’ OR ‘Operative Surgical Procedure*’ OR ‘Procedure*, Operative Surgical’ OR ‘Surgical Procedure*, Operative’ OR ‘General Surgery’ OR ‘Surgery, General’ OR ‘Surgery’ OR ‘Surgical’ OR ‘Surg’ OR ‘Operation’ OR ‘invasive procedure*’ OR ‘operative therap*’ OR ‘operative procedure*’ OR ‘procedure* *operative’ OR ‘procedure’ OR ‘transplant*’ OR ‘*section’ OR ‘*otomy’ OR ‘*ectomy’ OR ‘*scopy’ OR ‘*plasty’ OR ‘replacement’ OR ‘debridement’ OR ‘graft’ OR ‘repair’ OR ‘reconstruction’ OR ‘fusion’ OR ‘fixation’ OR ‘reduction’ OR ‘release’ OR ‘decompression’)**ti,ab,kw**

**OR**

MeSH term: “*Surgical Procedure, Operative”*

**OR**

MeSH term: *“General Surgery”*

**Ovid Medline Search Strategy**

(‘Pneumonia*’ OR ‘pneumonitis’ OR ‘Pneumonitides’ OR ‘Pulmonary Inflammation’ OR ‘Inflammation, Pulmonary’ OR ’Lung Inflammation’ OR ‘Inflammation, Lung’ OR ‘Respiratory tract infection’ OR ‘Bronchopneumonia’ OR ‘Lobar Pneumonia’ OR ‘Post-Operative Complication*’ OR ‘Postoperative complication’ OR ‘Complication*, Post-Operative’ OR ‘Complication*, Postoperative’ OR ‘infective complication*’ OR ‘complication*, infective’ OR ‘pulmonary complication*’ OR ‘complication* pulmonary’ OR ‘postoperative pulmonary complication*’ OR ‘complication*, postoperative pulmonary’ OR ‘post-operative pulmonary complication*’ OR ‘complication*, post-operative pulmonary’ OR ‘PPC’)**abstract**

**OR**

(‘Pneumonia*’ OR ‘pneumonitis’ OR ‘Pneumonitides’ OR ‘Pulmonary Inflammation’ OR ‘Inflammation, Pulmonary’ OR ’Lung Inflammation’ OR ‘Inflammation, Lung’ OR ‘Respiratory tract infection’ OR ‘Bronchopneumonia’ OR ‘Lobar Pneumonia’ OR ‘Post-Operative Complication*’ OR ‘Postoperative complication’ OR ‘Complication*, Post-Operative’ OR ‘Complication*, Postoperative’ OR ‘infective complication*’ OR ‘complication*, infective’ OR ‘pulmonary complication*’ OR ‘complication* pulmonary’ OR ‘postoperative pulmonary complication*’ OR ‘complication*, postoperative pulmonary’ OR ‘post-operative pulmonary complication*’ OR ‘complication*, post-operative pulmonary’ OR ‘PPC’)**title**

**OR**

MeSH term: *“Pneumonia”*

**AND**

(‘biomarker*‘ OR ‘Marker* Biologic*‘ OR ‘Biologic* Marker*’ OR ‘Marker* Immun*’ OR ‘Immun* Marker*’ OR ‘Serum Marker*’ OR ‘Marker* Serum’ OR ‘Surrogate End point*’ OR ‘Endpoint* Surrogate*’ OR ‘Surrogate endpoint*’ OR ‘End Point*, Surrogate’ OR ‘Marker* Clinical’ OR ‘Clinical Marker*’ OR ‘Viral Marker*’ OR ‘Marker* Viral’ OR ‘biochemical marker*’ OR ‘marker biochemical*’ OR ‘laboratory marker*’ OR ‘marker* laboratory’ OR ‘surrogate marker*’ OR ‘marker* surrogate’ OR ‘blood marker*’ OR ‘marker* blood’)**abstract**

**OR**

(‘biomarker*‘ OR ‘Marker* Biologic*‘ OR ‘Biologic* Marker*’ OR ‘Marker* Immun*’ OR ‘Immun* Marker*’ OR ‘Serum Marker*’ OR ‘Marker* Serum’ OR ‘Surrogate End point*’ OR ‘Endpoint* Surrogate*’ OR ‘Surrogate endpoint*’ OR ‘End Point*, Surrogate’ OR ‘Marker* Clinical’ OR ‘Clinical Marker*’ OR ‘Viral Marker*’ OR ‘Marker* Viral’ OR ‘biochemical marker*’ OR ‘marker biochemical*’ OR ‘laboratory marker*’ OR ‘marker* laboratory’ OR ‘surrogate marker*’ OR ‘marker* surrogate’ OR ‘blood marker*’ OR ‘marker* blood’)**title**

**OR**

MeSH term: *“Biomarkers”*

**AND**

(‘Surgical Procedure*, Operative’ OR ‘Surgical Procedure’ OR ‘Procedure*, Surgical’ OR ‘Operative Procedure*’ OR ‘Procedure*, Operative’ OR ‘Operative Surgical Procedure*’ OR ‘Procedure*, Operative Surgical’ OR ‘Surgical Procedure*, Operative’ OR ‘General Surgery’ OR ‘Surgery, General’ OR ‘Surgery’ OR ‘Surgical’ OR ‘Surg’ OR ‘Operation’ OR ‘invasive procedure*’ OR ‘operative therap*’ OR ‘operative procedure*’ OR ‘procedure* *operative’ OR ‘procedure’ OR ‘transplant*’ OR ‘*section’ OR ‘*otomy’ OR ‘*ectomy’ OR ‘*scopy’ OR ‘*plasty’ OR ‘replacement’ OR ‘debridement’ OR ‘graft’ OR ‘repair’ OR ‘reconstruction’ OR ‘fusion’ OR ‘fixation’ OR ‘reduction’ OR ‘release’ OR ‘decompression’) **abstract**

**OR**

(‘Surgical Procedure*, Operative’ OR ‘Surgical Procedure’ OR ‘Procedure*, Surgical’ OR ‘Operative Procedure*’ OR ‘Procedure*, Operative’ OR ‘Operative Surgical Procedure*’ OR ‘Procedure*, Operative Surgical’ OR ‘Surgical Procedure*, Operative’ OR ‘General Surgery’ OR ‘Surgery, General’ OR ‘Surgery’ OR ‘Surgical’ OR ‘Surg’ OR ‘Operation’ OR ‘invasive procedure*’ OR ‘operative therap*’ OR ‘operative procedure*’ OR ‘procedure* *operative’ OR ‘procedure’ OR ‘transplant*’ OR ‘*section’ OR ‘*otomy’ OR ‘*ectomy’ OR ‘*scopy’ OR ‘*plasty’ OR ‘replacement’ OR ‘debridement’ OR ‘graft’ OR ‘repair’ OR ‘reconstruction’ OR ‘fusion’ OR ‘fixation’ OR ‘reduction’ OR ‘release’ OR ‘decompression’) **title**

**Embase Search Strategy**

(‘Pneumonia*’ OR ‘pneumonitis’ OR ‘Pneumonitides’ OR ‘Pulmonary Inflammation’ OR ‘Inflammation, Pulmonary’ OR ’Lung Inflammation’ OR ‘Inflammation, Lung’ OR ‘Respiratory tract infection’ OR ‘Bronchopneumonia’ OR ‘Lobar Pneumonia’ OR ‘Post-Operative Complication*’ OR ‘Postoperative complication’ OR ‘Complication*, Post-Operative’ OR ‘Complication*, Postoperative’ OR ‘infective complication*’ OR ‘complication*, infective’ OR ‘pulmonary complication*’ OR ‘complication* pulmonary’ OR ‘postoperative pulmonary complication*’ OR ‘complication*, postoperative pulmonary’ OR ‘post-operative pulmonary complication*’ OR ‘complication*, post-operative pulmonary’ OR ‘PPC’)**abstract**

**OR**

(‘Pneumonia*’ OR ‘pneumonitis’ OR ‘Pneumonitides’ OR ‘Pulmonary Inflammation’ OR ‘Inflammation, Pulmonary’ OR ’Lung Inflammation’ OR ‘Inflammation, Lung’ OR ‘Respiratory tract infection’ OR ‘Bronchopneumonia’ OR ‘Lobar Pneumonia’ OR ‘Post-Operative Complication*’ OR ‘Postoperative complication’ OR ‘Complication*, Post-Operative’ OR ‘Complication*, Postoperative’ OR ‘infective complication*’ OR ‘complication*, infective’ OR ‘pulmonary complication*’ OR ‘complication* pulmonary’ OR ‘postoperative pulmonary complication*’ OR ‘complication*, postoperative pulmonary’ OR ‘post-operative pulmonary complication*’ OR ‘complication*, post-operative pulmonary’ OR ‘PPC’)**title**

**OR**

MeSH term: *“Pneumonia”*

**AND**

(‘biomarker*‘ OR ‘Marker* Biologic*‘ OR ‘Biologic* Marker*’ OR ‘Marker* Immun*’ OR ‘Immun* Marker*’ OR ‘Serum Marker*’ OR ‘Marker* Serum’ OR ‘Surrogate End point*’ OR ‘Endpoint* Surrogate*’ OR ‘Surrogate endpoint*’ OR ‘End Point*, Surrogate’ OR ‘Marker* Clinical’ OR ‘Clinical Marker*’ OR ‘Viral Marker*’ OR ‘Marker* Viral’ OR ‘biochemical marker*’ OR ‘marker biochemical*’ OR ‘laboratory marker*’ OR ‘marker* laboratory’ OR ‘surrogate marker*’ OR ‘marker* surrogate’ OR ‘blood marker*’ OR ‘marker* blood’)**abstract**

**OR**

(‘biomarker*‘ OR ‘Marker* Biologic*‘ OR ‘Biologic* Marker*’ OR ‘Marker* Immun*’ OR ‘Immun* Marker*’ OR ‘Serum Marker*’ OR ‘Marker* Serum’ OR ‘Surrogate End point*’ OR ‘Endpoint* Surrogate*’ OR ‘Surrogate endpoint*’ OR ‘End Point*, Surrogate’ OR ‘Marker* Clinical’ OR ‘Clinical Marker*’ OR ‘Viral Marker*’ OR ‘Marker* Viral’ OR ‘biochemical marker*’ OR ‘marker biochemical*’ OR ‘laboratory marker*’ OR ‘marker* laboratory’ OR ‘surrogate marker*’ OR ‘marker* surrogate’ OR ‘blood marker*’ OR ‘marker* blood’)**title**

**OR**

MeSH term: *“Biomarkers”*

**AND**

(‘Surgical Procedure*, Operative’ OR ‘Surgical Procedure’ OR ‘Procedure*, Surgical’ OR ‘Operative Procedure*’ OR ‘Procedure*, Operative’ OR ‘Operative Surgical Procedure*’ OR ‘Procedure*, Operative Surgical’ OR ‘Surgical Procedure*, Operative’ OR ‘General Surgery’ OR ‘Surgery, General’ OR ‘Surgery’ OR ‘Surgical’ OR ‘Surg’ OR ‘Operation’ OR ‘invasive procedure*’ OR ‘operative therap*’ OR ‘operative procedure*’ OR ‘procedure* *operative’ OR ‘procedure’ OR ‘transplant*’ OR ‘*section’ OR ‘*otomy’ OR ‘*ectomy’ OR ‘*scopy’ OR ‘*plasty’ OR ‘replacement’ OR ‘debridement’ OR ‘graft’ OR ‘repair’ OR ‘reconstruction’ OR ‘fusion’ OR ‘fixation’ OR ‘reduction’ OR ‘release’ OR ‘decompression’) **abstract**

**OR**

(‘Surgical Procedure*, Operative’ OR ‘Surgical Procedure’ OR ‘Procedure*, Surgical’ OR ‘Operative Procedure*’ OR ‘Procedure*, Operative’ OR ‘Operative Surgical Procedure*’ OR ‘Procedure*, Operative Surgical’ OR ‘Surgical Procedure*, Operative’ OR ‘General Surgery’ OR ‘Surgery, General’ OR ‘Surgery’ OR ‘Surgical’ OR ‘Surg’ OR ‘Operation’ OR ‘invasive procedure*’ OR ‘operative therap*’ OR ‘operative procedure*’ OR ‘procedure* *operative’ OR ‘procedure’ OR ‘transplant*’ OR ‘*section’ OR ‘*otomy’ OR ‘*ectomy’ OR ‘*scopy’ OR ‘*plasty’ OR ‘replacement’ OR ‘debridement’ OR ‘graft’ OR ‘repair’ OR ‘reconstruction’ OR ‘fusion’ OR ‘fixation’ OR ‘reduction’ OR ‘release’ OR ‘decompression’) **title**

**OR**

MeSH term: *“Surgical”*

**OR**

MeSH term: *“general surgery”*
