# Supplementary material for: Biomarkers associated with post-operative pneumonia: a systematic review and meta-analysis
Source: BMC Pulm Med. 2026 Jul 16;26:455. doi: 10.1186/s12890-026-04486-7 (PMC13651040; doi:10.1186/s12890-026-04486-7)
Supplement: Supplementary file 3 [file 12890_2026_4486_MOESM3_ESM.docx]

**Additional File 3**

Clinical Diagnostic Criteria for Post-Operative Pneumonia

The diagnostic criteria used for each included study, with respective references, the time of pneumonia assessment and pneumonia diagnosis. When not recorded by the authors, abbreviation NR is listed in the table below.

| Study | Time of Assessment | Time of Diagnosis | Reported Pneumonia Diagnosis Criteria | Listed Reference |
| --- | --- | --- | --- | --- |
| As, 2025 | NR | 3.4 ± 2.6 days post-op | In patients with clinically suspected pneumonia, the presence of newly detected infiltration on chest X-ray or an increase in the degree of existing infiltration was considered indicative of pneumonia. In addition to at least two of the following:  1) Fever (>38.5 °C) or hypothermia (<36.0 °C); 2) Presence of purulent tracheobronchial secretions or an increase in the quantity of existing secretions; and 3) Leukocytosis (≥12,000/μL) or leukopenia (≤4000/μL). C-reactive protein and procalcitonin levels were also utilized to support the diagnosis. | Köse A, Yurtseven N, Düzyol IY. Ventilator associated pneumonia after open heart surgery: risk factors. GKDA Derg 2019;25(3):181–9. https://doi.org/10.5222/GKDAD.2019.00719. |
| Averdunk, 2020 | NR | NR | According to the guidelines of the American Thoracic Society. | American Thoracic, S., Infectious Diseases Society of, A. Guidelines for the management of adults with hospital-acquired, ventilator-associated, and healthcare-associated pneumonia. *Am. J. Respir. Crit. Care Med.* 171, 388–416(2005) |
| Chen, 2022 | Within the first 5 days post-op | NR | 1) Clinical: The presence of a new or progressive radiographic infiltrate plus at least two; Fever greater than 38 °C; Leukocytosis or leucopenia; Purulent secretions  2) Bacteriological: Sputum culture was performed at least twice with fiberoptic bronchoscope or alveolar lavage fluid; Pathogenic bacteria were detected in sputum culture; The first positive result appeared within 48 h after surgery  The diagnosis was determined by two independent experts based on complete clinical records, with a third expert to reach consensus in cases of disagreement. | 1) Ailawadi G, et al. Pneumonia after cardiac surgery: experience of the national institutes of health/Canadian institutes of health research cardiothoracic surgical trials network. J Thorac Cardiovasc Surg. 2017;153(6):1384-1391.e3.  2) Kalil AC, et al. Management of adults with hospital-acquired and ventilator-associated pneumonia: 2016 clinical practice guidelines by the infectious diseases society of America and the American thoracic society. Clin Infect Dis. 2016;63(5):e61–111. |
| Chen, 2023 | Within 30-days post-op | NR | Categorised using a modified Clavien-Dindo Classification. | Dindo D, Demartines N, Clavien PA. Classification of surgical complications: a new proposal with evaluation in a cohort of 6336 patients and results of a survey. *Ann Surg* 2004;240:205-13. |
| de la Varga-Martinez, 2022 | NR | NR | According to the Centres for Disease Control and Prevention definitions for pulmonary infection. | Garner, J.S.; Jarvis, W.R.; Emori, T.G.; Horan, T.C.; Hughes, J.M. CDC definitions for nosocomial infections. *Am. J. Infect. Control* 1988, *16*, 128–140. |
| Desmard, 2015 | Up to ICU discharge | Median of 8 (range 2 - 21) days post-op | Fiberoptic bronchoscopy was performed daily.  Lower respiratory tract infection was suspected on the basis of; New or persistent lung infiltrate on chest X-ray; Temperature >38.3^o^C or <36^o^C; Increased white blood cell count; Decreased PaO2/FIO2 ratio; Purulent endotracheal aspirate or sputum.  Pneumonia was diagnosed on the basis of the results of microbiologic culture of bronchial samples yielding >10^6^ colony-forming units (CFU)/ml. | NR |
| Ding, 2025 | Postoperative pneumonia within 30 days of surgery | NR | New or progressive pulmonary infiltrates on chest radiography/CT plus evidence of systemic inflammatory response (leucocytosis >12×109/L or leukopenia <4×109/L with fever >38 °C and/or altered mental status) and ≥2 clinical features (e.g., purulent sputum, change in sputum characteristics, increased secretions requiring suctioning, or dyspnoea). All suspected cases were adjudicated by two clinicians. | “As per CDC/NHSN criteria” |
| Ishida, 2024 | NR | Median day 4 (range 1-12 days) | Radiographs were obtained daily from POD 1 to 7. In addition, if high inflammation levels were indicated in blood tests and/or respiratory symptoms were present, computed tomography (CT) was performed to confirm the presence of pneumonia. | Clavien–Dindo grades ≥ 2 |
| Ji, 2021 | Within 30-days after surgery | NR | - New and persistent pulmonary infiltrate on chest X-ray and/or CT, and one or more of the following criteria: - Presence of new and/or progressive and steady clinical symptoms, including cough, dyspnoea or expectoration - Moist rale and/or lung consolidation by clinical examination - Body temperature abnormalities (>38°C or <36.0°C) - WBC abnormalities (white cell count >10×10^9^ /L or <4×10^9^ /L) - Positive bacterial culture of blood samples or sputum samples. | 1) Bohl DD, Sershon RA, Saltzman BM, et al. Incidence, risk factors, and clinical implications of pneumonia after surgery for geriatric hip fracture. J Arthroplasty 2018;33:1552–6.  2) Lv H, Yin P, Long A, et al. Clinical characteristics and risk factors of postoperative pneumonia after hip fracture surgery: a prospective cohort study. Osteoporos Int 2016;27:3001–9.  3) Wang Y, Li X, Ji Y, et al. Preoperative serum albumin level as a predictor of postoperative pneumonia after femoral neck fracture surgery in a geriatric population. Clin Interv Aging 2019;14:2007–16. |
| Jiao, 2015 | 48 hours to 6-days after initiation of ventilation | NR | - Chest X-ray results, with at least two of the following features: - Fever with a body temperature of >38 ̊C; - White blood cell count of >11,000 or <3,000/μl - Presence of purulent endotracheal secretions. - Microbiological samples were obtained by fibroscopic BAL and the growth of ≥104 colony-forming units/ml microorganism culture. | NR |
| Jin, 2021 | Within 5 days after surgery | NR | - Microorganisms were isolated from bronchial secretions at least twice (the first microorganism obtained on POD2) and at least one of the following: - Purulent sputum - Auscultation findings suspicious of pneumonia - Hypoxemia - Body temperature >38°C - and infiltrate on chest radiograph   The final diagnosis was determined by 2 independent experts in regard to the complete medical chart. In cases of disagreement, a third expert helped reach a consensus. | 1) Bone RC, Balk RA, Cerra FB, et al. Definitions for sepsis and organ failure and guidelines for the use of innovative therapies in sepsis. The ACCP/SCCM Consensus Conference Committee. American College of Chest Physicians/Society of Crit Care Med. Chest 1992;101:1644-1655.  2) Calandra T, Cohen J. The International Sepsis Forum Consensus Conference on Definitions of Infection in the Intensive Care Unit. Crit Care Med 2005;33:1538-1548. |
| Li, 2021 | NR | NR | According to the diagnostic criteria published by the Centers for Disease Control and Prevention/National Healthcare Safety Network (CDC/NHSN)  RADIOLOGY: Two or more serial chest radiographs with at least one of the following:   - New or progressive and persistent infiltrate - Consolidation - Cavitation   SIGNS/SYMPTOMS: At least one of the following:   - Fever (>38°C) with no other recognized cause - Leukopenia (<4000 WBC/mm^3^) or leukocytosis (≥12,000 WBC/mm^3^) - For adults ≥70 years, altered mental status with no other recognized cause and at least two of the following: - New onset of purulent sputum or change in character of sputum or increased respiratory secretions or increased suctioning requirements - New onset or worsening cough, or dyspnea, or tachypnea - Rales or bronchial breath sounds - Worsening gas exchange (eg, O2 desaturations [eg, PaO2/FiO2 ≤240], or increased oxygen requirements, or increased ventilator demand). | Horan TC, Andrus M, Dudeck MA. 2008. CDC/NHSN surveillance def- inition of health care-associated infection and criteria for specific types of infections in the acute care setting. Am J Infect Control. 36(5):309-332. |
| Li F, 2025 | NR | NR | POP was diagnosed based on the presence of at least three of the following features: (1) lung exudation and consolidation on chest radiography or CT, (2) fever (>38 ◦C), (3) WBC count of >10,000/mm3 or <3000/mm3, and (4) opportunistic pathogens in the sputum or bronchial secretions obtained by bronchoscopy. | Jiang R, Li P, Shen W, et al. The predictive value of the preoperative systemic immune-inflammation index in the occurrence of postoperative pneumonia in non- small cell lung cancer: a retrospective study based on 1486 cases. Thorac Cancer. 2023 Jan;14(1):30–35. https://doi.org/10.1111/1759-7714.14691. Epub 2022 Dec 9. PMID: 36495040; PMCID: PMC9807440. |
| Li J, 2025 | NR | NR | Determined by attending physicians, radiologists, and laboratory physicians. Additionally, samples of sputum, wound secretions, whole blood, urine, or locked punctures were collected for bacterial culture and LAMP joint testing. | Criteria for “Pneumonia Diagnosis” (WS 382–2012) but not referenced |
| Lin, 2024 | NR | Median day 10 (range 2 - 18 days) | Patients with VAP should meet the standard diagnostic and treatment guidelines for CAP or the diagnostic criteria for HAP formulated by the Respiratory Diseases Branch of the Chinese Medical Association in 2006. Diagnostic basis: (1) new cough, sputum, and increased respiratory secretions such as pus sputum with or without chest pain; (2) fever (>37.5°C); (3) physical examination: signs of pulmonary solid changes and/or wet rales in the lungs on auscultation; (4) in the peripheral blood, WBC was >10x109/L or <4x109/L with or without leftward nuclear shift; (5) imaging: CXR or CT examination suggest lungs have flaky, patchy, or mass infiltrative imaging changes or interstitial changes with or without pleural effusion. Add item 5 to any one of the above 1–4 items, and make a diagnosis of VAP after excluding diseases such as lung cancer, pulmonary oedema, aspiration or foreign body aspiration, pulmonary tuberculosis, pulmonary infarction, and non‐infectious pulmonary interstitial disease. | Respiratory Diseases Branch of the Chinese Medical Association in 2006 |
| Liu, 2015 | Within 4 days | NR | With mechanical ventilation >48 h’ duration and the presence of;   - New and/ or progressive pulmonary infiltrates on a chest radiograph plus two or more of the following criteria of intubation and mechanical ventilation; - Fever (≥38.5 °C) or hypothermia (36 °C) - Blood leucocytosis (>12 × 10^9^ white blood cells/L) or leucopenia (<4 × 10^9^ white blood cells/L) - Purulent tracheobronchial secretions - The isolation of one or more pathogenic microorganisms in significant bacterial counts was required to confirm the diagnosis of VAP | NR |
| Liu, 2019 | Within 3 days | NR | - Chest roentgenogram change (new or progressive and persistent infiltration; consolidation; or cavitation) within 72 hours post op, and 1 or more of below: - Fever (>38°C) with no other recognized cause - Leucopenia (<4000 WBCs/mm^3^) or leukocytosis (>12 000 WBCs/mm^3^) - Purulent secretion - Positive bacteriology tests for sputum. | 1) Perrotti A, Chenevier-Gobeaux C, Ecarnot F et al: Is endocan a diagnostic marker for pneumonia after cardiac surgery? The ENDOLUNG Study. Ann Thorac Surg, 2018; 105(2): 535–41  2) Garner JS, Jarvis WR, Emori TG et al: CDC definitions for nosocomial infec- tions, 1988. Am J Infect Control, 1988; 16(3): 128–40 |
| Lv, 2016 | NR | NR | - New infiltrations in their chest X-ray postoperatively and one or more below; - Presence of new and/or progressive and persistent respiratory symptoms including coughing and expectoration - Presence of fever or hypothermia - Physical examination showing lung consolidation signs and/or moist rale - White cell count of greater than 10 × 10^9^/L or less than 4 × 10^9^/L, - Pathogen isolation from blood culture or sputum. | 1) Cakir Edis E, Hatipoglu ON, Yilmam I, Eker A, Tansel O, Sut N (2009) Hospital-acquired pneumonia developed in non-intensive care units. Respiration 78:416–422.  2) American Thoracic S, Infectious Diseases Society of A (2005) Guidelines for the management of adults with hospital-acquired, ventilator-associated, and healthcare-associated pneumonia. Am J Respir Crit Care Med 171:388–416 .  3) Kieninger AN, Lipsett PA (2009) Hospital-acquired pneumonia: pathophysiology, diagnosis, and treatment. Surg Clin North Am 89:439–461 |
| Mao, 2025 | Within 7 days post op | NR | (1) Presence of at least one of the following on a minimum of two chest radiographs (only one chest radiograph is necessary for patients lacking underlying cardiopulmonary disease): (a) New or progressive persistent infiltrative shadows; (b) Solid lesions; (c) Cavitation; (2) Fulfilment of at least one of the following criteria: (a) Unexplained fever > 38 °C; (b) WBC count decreased (<4×10^9/L) or increased (>12×10^9/L); (c) Unexplained change in mental status in adults aged 70 years or older; (3) At least two of the following criteria are met: (a) New onset of purulent sputum, change in sputum character, increase in respiratory secretions, or increased need for suctioning; (b) New or worsening cough, dyspnoea, or shortness of breath; (c) Lung rales or bronchial breath sounds; (d) Deterioration in gas exchange function (hypoxemia, increased oxygen demand, or increased ventilator requirements). The results of pathogenic cultures serve as a foundational basis for diagnosing POP. | 2018 CDC diagnostic criteria for pneumonia  Iosifidis E, Pitsava G, Roilides E. Ventilator-Associated pneumonia in neonates and children: A systematic analysis of diagnostic methods and prevention. Future Microbiol. 2018;13:1431–46.  Abbott TEF, Fowler AJ, Pelosi P, Gama de Abreu M, Møller AM, Canet J, et al. A systematic review and consensus definitions for standardised end- points in perioperative medicine: pulmonary complications. Br J Anaesth. 2018;120(5):1066–79. |
| Ozawa, 2026 | NR | NR | Postoperative complications according to the Clavien–Dindo classification of surgical complications and results of neo- adjuvant chemotherapy (NAC) were also collected. | NR |
| Perrotti, 2018 | NR | Average time to diagnosis of POP was 96 +/- 60 hours | - New detection of lung infiltrates on chest radiograph associated with at least two of the following: - Fever more than 38.8C - Leukocytosis (>11,000 cells/mm^3^) or leucopenia (<3,000 cells/mm^3^ ) - Purulent secretion - Confirmed by microbiologic analysis using BAL with fiberoptic bronchoscopy. | Garner JS, Jarvis WR, Emori TG, et al. CDC definitions for nosocomial infections, 1988. Am J Infect Control 1988;16: 128–40. |
| Ruan, 2025 | NR | NR | The diagnosis of POP was based on the presence of at least three of the following features: (1) lung exudation and consolidation on chest radiographs or CT scans, (2) fever (> 38°C), (3) WBC count > 10000/mm3 or < 3000/mm3, (4) opportunistic pathogens in the sputum or bronchial secretions obtained by bronchoscopy | Jiang R, Li P, Shen W, Deng H, Qin C, Qiu X, et al. The predictive value of the preoperative systemic immune-inflammation index in the occurrence of postoperative pneumonia in non-small cell lung cancer: A retrospective study based on 1486 cases. Thorac Cancer. (2023) 14:30–5. doi: 10.1111/1759-7714.14691 |
| Tang, 2024 | Within 30 days post op | NR | POP was defined based on diagnostic criteria from the American College of Chest Physicians (ACCP) and European Respiratory Society (ERS). A POP diagnosis required meeting one or more of the following criteria: (1) the emergence or exacerbation of respiratory symptoms, such as cough and purulent discharge; (2) a body temperature exceeding 38 °C or below 36.0 °C; (3) the identification of lung consolidation or the presence of crackles during a physical examination; (4) an abnormal white blood cell count indicative of either leukocytosis (> 10 × 109/L) or leukopenia (< 4 × 109/L); (5) the confirmation of relevant pathogens through microbial culture in sputum or blood samples. Two blinded respiratory physicians identified POP through individual chart reviews. Any conflicts were resolved by a third respiratory physician. | Harrington, D. et al. New guidelines for statistical reporting in the journal. New Engl. J. Med. 381, 285–286. https://doi.org/10. 1056/NEJMe1906559 (2019).  American College of Chest Physicians (ACCP) and European Respiratory Society (ERS |
| Singh, 2023 | NR | median value of POD 5 | - Purulent sputum - Fever of more than 38°C - White blood cell count of more than 11,000 or less than 3,000/mm^3^, - Fresh pulmonary infiltrates on a Chest X-Ray | Takakura Y, Hinoi T, Egi H, Shimomura M, Adachi T, Saito Y, et al. Procalcitonin as a predictive marker for surgical site infection in elective colorectal cancer surgery. Langenbecks Arch Surg. 2013;398:833–839. |
| Song, 2020 | NR | NR | - Use of mechanical ventilation for more than 48 h, or the removal of the oxygenator and extubation within 48h. - New or progressively enlarged infiltrative shadows in the lungs. - Signs of pulmonary consolidation and/or moist rales, and one of the below: - White blood cell count was more than 10x10^9^/L or less than 4x10^9^/L; - With or without a nuclear shift to the left - Body temperature >38C or <36C - Purulent secretion in the respiratory tract - New pathogens were isolated from bronchial secretions after onset. | 1 )Kunst G, Milojevic M, Boer C, et al. 2019 EACTS/EACTA/EBCP guidelines on cardiopulmonary bypass in adult cardiac surgery. Br J Anaesth. 2019;123:713–757.  2) Monaco F, Di Prima AL, De Luca M, et al. Periprocedural and perioperatory management of patients with tricuspid valve disease. Minerva Cardioangiol. 2018;66(6):691–699. |
| Toh Yoon, 2019 | NR | NR | NR | NR |
| Wang, 2020 | NR | NR | - Presence of new respiratory symptoms such as coughing and expectoration - Progressive chronic respiratory symptoms including coughing, expectoration, and shortness of breath - Fever or hypothermia - New infiltrations observed in chest X-rays or computed tomography. | NR |
| Wen, 2025 | Daily assessment throughout the hospital stay. | NR | Daily clinical assessments included vital sign measurements every 4 h, chest auscultation twice daily, daily evaluation of sputum characteristics, and continuous oxygen saturation monitoring. Chest X-rays were performed on postoperative day 1 and as clinically indicated thereafter. Blood tests, including complete blood count and C-reactive protein, were conducted on postoperative days 1, 3, and 5, and as needed based on clinical suspicion of infection.  Patients were diagnosed with HAP based on the following criteria: (1) new onset of productive cough with purulent sputum, with or without chest pain; (2) fever (body temperature > 37.5°C); (3) physical examination findings of lung consolidation and/or moist rales; (4) peripheral blood white blood cell count >10 × 109/L or <4 × 109/L, with or without a left shift; (5) imaging studies showing new infiltrates, consolidation, or interstitial patterns on chest X-ray or CT scan, with or without pleural effusion. A diagnosis of pulmonary infection was established after excluding lung cancer, pulmonary edema, aspiration, foreign body inhalation, tuberculosis, pulmonary embolism, and non-infectious interstitial lung diseases, based on at least one of the first four criteria in conjunction with the fifth criterion. | NR |
| White, 2011 | Day 2 to day 5 post-op | NR | Based on the Centers for Disease Control and Prevention definition | Centers for Disease Control and Prevention. CDC definitions for nosocomial infections, 1988. Am Rev Respir Dis. 1989;139(4):1058-1059. |
| Wu, 2025 | 24 hours post op until discharge | NR | POP was diagnosed according to the American Thoracic Society guidelines for healthcare-associated pneumonia. The criteria included (1) the presence of new and/or progressive and persistent respiratory symptoms, such as cough; (2) fever or hypothermia; (3) lung consolidation and auscultatory crackles; (4) WBC > 10 × 109 cells/L or WBC < 4 × 109 cells/L; and (5) new infiltrations observed on chest X-ray or computed tomography. Diagnosis required meeting criteria 1–4 in con- junction with criterion 5 within 24h postoperatively until discharge. | Kalil AC, Metersky ML, Klompas M, et al. Management of adults with hospital-acquired and ventilator-associated pneumonia: 2016 clinical practice guidelines by the Infectious Diseases Society of America and the American Thoracic Society. Clin Infect Dis. 2016;63 (5): e61–e111. doi: 10.1093/cid/ciw353 |
| Xie, 2022 | NR | NR | - Pathogenic bacteria were detected at least twice - New detection of lung infiltrates on chest radiograph associated with at least two of three clinical features: - Fever more than 38°C - Leukocytosis or leukopenia - Purulent secretion   The final diagnosis was determined by two independent experts in regard to the complete clinical records. In cases of disagreement between the two clinical experts, a third expert reached a consensus. The final diagnosis was reached when it was classified as microbiologically confirmed by the experts and ruled out when the experts classified it as absent, probable, or possible. | 1) Kalil AC, Metersky ML, Klompas M, Muscedere J, Sweeney DA, Palmer LB, et al. 2016. Management of Adults With Hospital-acquired and Ventilator-associated Pneumonia: 2016 Clinical Practice Guidelines by the Infectious Diseases Society of America and the American Thoracic Society. Clinical infectious diseases : an official publication of the Infectious Diseases Society of America. 63(5):e61-111.  2) Calandra T, Cohen J. 2005. The international sepsis forum consen- sus conference on definitions of infection in the intensive care unit. p. 1538-48. |
| Yao, 2023 | 24 hours up to discharge | NR | - New infiltrates on postoperative chest X-rays or chest computed tomography, confirmed through consultation with a respiratory physician - Criteria outlined in the American Thoracic Society guidelines for HAP: - Presence of new and/or progressive and persistent respiratory symptoms, such as cough and purulent secretions - Postoperative fever (> 38.0 °C) or hypothermia (< 36.0 °C) - Physical examinations revealing lung consolidation and auscultatory crackles - White blood cell count that exceeds 10 × 10^9^/L or falls below 4 × 10^9^/L - Patchy inflammatory shadows or interstitial changes on chest X-rays. - Provided that lung cancer, tuberculosis, pulmonary embolism, and other pulmonary diseases have been ruled out. | 1) Ahn J, Chang JS, Kim JW. Postoperative pneumonia and aspiration pneu- monia following elderly hip fractures. J Nutr Health Aging. 2022;26:732–8. https://doi.org/10.1007/s12603-022-1821-9.  2) Kalil AC, Metersky ML, Klompas M, Muscedere J, Sweeney DA, Palmer LB, Napolitano LM, O’Grady NP, Bartlett JG, Carratalà J, El Solh AA, Ewig S, Fey PD, File TM Jr, Restrepo MI, Roberts JA, Waterer GW, Cruse P, Knight SL, Brozek JL. Management of adults with hospital-acquired and ventilator- associated pneumonia: 2016 clinical practice guidelines by the Infectious Diseases Society of America and the American Thoracic Society. Clin Infect Dis Off Publ Infect Dis Soc Am. 2016;63:e61–111. https://doi.org/10. 1093/cid/ciw353. |
| Yuan, 2022 | Within 30 days after surgery | NR | - Fever - Increased WBC and C-reactive protein (CRP) levels - Chest radiograph showed pulmonary infiltrates - Required antibiotic therapy by a surgeon, according to the modified Centers for Disease Control and Prevention criteria | Smith CJ, Kishore AK, Vail A, Chamorro A, Garau J, Hopkins SJ, et al. Diagnosis of stroke-associated pneumonia: recommendations from the pneumonia in stroke consensus group. Stroke. (2015) 46:2335– 40. doi: 10.1161/STROKEAHA.115.009617 |
| Zhang, 2022 | Within 30 days after surgery | NR | According to the modified Centers for Disease Control and Prevention (CDC) criteria   - Not diagnosed on admission and not explained by another diagnosis - Confirmed change in diagnostic on at least one image of the chest X-ray. | Smith CJ, Kishore AK, Vail A, Chamorro A, Garau J, Hopkins SJ, et al. Diagnosis of stroke-associated pneumonia: recommendations from the pneumonia in stroke consensus group. Stroke. (2015) 46:2335. doi: 10.1161/STROKEAHA.115.009617 |
| Zhao, 2022 | NR | NR | The improved Centres for Disease Control and Prevention (CDC) criteria was used:   - Fever (temperature > 38 °C) with no other recognized cause - Leukopenia (< 4 × 10^9^/L) or leukocytosis (> 12 × 10^9^/L), - Age > 70 years, altered mental status with no other recognized cause - New onset of purulent sputum, change in character of sputum over a 24 h period, increased respiratory secretions, or increased suctioning, - New or worsening cough, dyspnoea, or shortness of breath (respiratory rate>25/min), - Rales, crackles, or bronchial breath sounds - Gas exchange disorders (hypoxemia: PaO2/FiO2 ≤ 240; increased oxygen). - Diagnosis also required pulmonary CT appearance or progressive infiltration, consolidation, or ground glass | Smith, C .J. et al. Diagnosis of stroke-associated pneumonia: Recommendations from the pneumonia in Stroke Consensus Group. Stroke 46, 2335–2340. https://doi.org/10.1161/strokeaha.115.009617 (2015). |
| Zhou, 2024 | NR | NR | Suspected pneumocystis jirovecii infection (with symptoms of lung infection and abnormal findings on chest images). The patients who had positive polymerase chain reaction (PCR) results of pneumocystis jirovecii and confirmed PJP diagnosis were enrolled in the study. | NR |
| Zuo, 2019 | Within 30 days post-surgery | NR | Radiological standards:   - Definitive chest X-ray or CT examination representing at least one of the following: new or progressive and consistent infiltration, opacity or consolidation, and cavitation.   Clinical symptoms:   - Existence new or progressive and continuous coughing and expectoration. - Fever (> 38 °C) with no other recognized cause - Abnormal WBC: leukopenia (<4000/mm^3^) or leukocytosis (≥12,000/mm^3^). - Positive respiratory cultures from sputum or blood. | 1) Kieninger AN, Lipsett PA. Hospital-acquired pneumonia: pathophysiology, diagnosis, and treatment. Surg Clin North Am. 2009;89:439–61.  2) Clavien PA, Barkun J, de Oliveira ML, Vauthey JN, Dindo D, Schulick RD, et al. The Clavien-Dindo classification of surgical complications: five-year experience. Ann Surg. 2014;250:187.  3) Niederman MS, Craven DE. Guidelines for the management of adults with hospital-acquired, ventilator-associated, and healthcare-associated pneumonia. Am J Respir Crit Care Med. 2005;171:388–416. |
