# Supplementary material for: Biomarkers associated with post-operative pneumonia: a systematic review and meta-analysis
Source: BMC Pulm Med. 2026 Jul 16;26:455. doi: 10.1186/s12890-026-04486-7 (PMC13651040; doi:10.1186/s12890-026-04486-7)
Supplement: Supplementary file 4 [file 12890_2026_4486_MOESM4_ESM.docx]

**Additional File 4.**

**Additional File 4 Table:** Pre-operative biomarkers reported in 1 study only.

| **Biomarker** | **SMD** | **LL** | **UL** | ***p*** | **Sample Size (n)** | |
| --- | --- | --- | --- | --- | --- | --- |
|  |  |  |  |  | POP | No POP |
| Lymphocyte % | -1.940 | -2.45 | -1.43 | **<0.0001** | 31 | 62 |
| Red Blood Cell Distribution Width | 0.787 | 0.55 | 1.03 | **<0.0001** | 70 | 1359 |
| CD8 T Cells | -1.286 | -1.76 | -0.82 | **<0.0001** | 31 | 62 |
| CD4 T Cells | -1.226 | -1.69 | -0.76 | **<0.0001** | 31 | 62 |
| Neutrophils % | 1.199 | 0.74 | 1.66 | **<0.0001** | 31 | 62 |
| CD3 T Cells | -1.085 | -1.54 | -0.63 | **<0.0001** | 31 | 62 |
| CD19 B Cells | -1.073 | -1.53 | -0.62 | **<0.0001** | 31 | 62 |
| Blood Concentration of Tacrolimus | -0.969 | -1.42 | -0.52 | **<0.0001** | 31 | 62 |
| NK Cell Count | -0.947 | -1.40 | -0.49 | **<0.0001** | 31 | 62 |
| Red Blood Cell Distribution width: coefficient of variation | -0.477 | -0.76 | -0.20 | **0.0009** | 51 | 1105 |
| Monocyte % | -0.682 | -1.12 | -0.24 | **0.002** | 31 | 62 |
| Toll-Like Receptor 4 (TLR4) Expression on Lymphocytes | 1.725 | 0.60 | 2.85 | **0.003** | 7 | 10 |
| Toll-Like Receptor 4 (TLR4) Expression on Monocytes | 1.643 | 0.53 | 2.76 | **0.004** | 7 | 10 |
| Prothrombin Time Activity | -0.596 | -1.04 | -0.16 | **0.008** | 31 | 62 |
| Serum Amyloid A | 0.332 | 0.08 | 0.59 | **0.01** | 93 | 172 |
| Alkaline Phosphate | 0.451 | 0.09 | 0.81 | **0.02** | 33 | 260 |
| Low Density Lipoprotein | -0.235 | -0.43 | -0.05 | **0.02** | 117 | 1162 |
| Creatinine Kinase-MB | -0.378 | -0.69 | -0.07 | **0.02** | 42 | 761 |
| eAG (estimated Average Glucose) | 0.302 | 0.05 | 0.55 | **0.02** | 71 | 478 |
| Red Blood Cell Distribution Width-Standard Deviation | 0.334 | 0.05 | 0.62 | **0.02** | 51 | 1105 |
| Neutrophilic Granulocyte % | 0.429 | 0.07 | 0.79 | **0.02** | 33 | 260 |
| International Normalised Ration (INR) | 0.500 | 0.06 | 0.94 | **0.02** | 31 | 62 |
| Lactate | 0.350 | 0.04 | 0.66 | **0.03** | 42 | 761 |
| sTREM-1 (Soluble Myeloid Triggering Receptor) | 0.281 | 0.03 | 0.53 | **0.03** | 93 | 172 |
| Indirect Bilirubin | -0.459 | -0.89 | -0.02 | **0.04** | 31 | 62 |
| Troponin T High Sensitivity | 0.319 | 0.01 | 0.63 | **0.04** | 42 | 761 |
| Endocan | -0.518 | -1.03 | -0.01 | **0.05** | 17 | 138 |
| Red Cell Distribution Width Percentage | 0.188 | 0.00 | 0.38 | 0.05 | 117 | 1162 |
| Secretory Leucocyte Peptidase Inhibitor | 0.828 | -0.08 | 1.73 | 0.07 | 6 | 27 |
| Toll-Like Receptor 4 | 0.212 | -0.04 | 0.46 | 0.10 | 93 | 172 |
| Gamma-Glutamyl Transpeptidase | 0.260 | -0.10 | 0.62 | 0.16 | 33 | 260 |
| High-Sensitivity C-Reactive Protein | 0.166 | -0.07 | 0.40 | 0.17 | 84 | 414 |
| Mean Corpuscular Haemoglobin | -0.192 | -0.47 | 0.09 | 0.18 | 51 | 1105 |
| Estimated Glomerular Filtration Rate | -0.330 | -0.84 | 0.18 | 0.20 | 17 | 138 |
| Magnesium | 0.180 | -0.13 | 0.49 | 0.26 | 42 | 761 |
| HbA1c | 0.143 | -0.11 | 0.39 | 0.26 | 71 | 478 |
| CD8 T Cells % | -0.216 | -0.65 | 0.22 | 0.33 | 31 | 62 |
| Mean Corpuscular Volume | -0.136 | -0.42 | 0.14 | 0.34 | 51 | 1105 |
| Neutrophil-to-Albumin Ratio | 0.120 | -0.13 | 0.37 | 0.34 | 78 | 317 |
| Creatine Phosphokinase | -0.135 | -0.45 | 0.18 | 0.39 | 42 | 761 |
| Plasma Fibrinogen | -0.084 | -0.33 | 0.16 | 0.51 | 78 | 317 |
| Erythrocytes | 0.095 | -0.22 | 0.41 | 0.55 | 42 | 761 |
| Messenger RNA IFN-y | 0.158 | -0.39 | 0.70 | 0.57 | 19 | 41 |
| Mean Platelet Volume | -0.053 | -0.24 | 0.14 | 0.59 | 117 | 1162 |
| CD3 T Cells % | 0.105 | -0.33 | 0.54 | 0.63 | 31 | 62 |
| Messenger RNA interleukin-23-p19 | -0.122 | -0.67 | 0.42 | 0.66 | 19 | 41 |
| IFN-y | -0.120 | -0.66 | 0.42 | 0.66 | 19 | 41 |
| Potential of Hydrogen (pH) | 0.055 | -0.22 | 0.33 | 0.69 | 55 | 846 |
| Messenger RNA Interleukin-10 | 0.105 | -0.44 | 0.65 | 0.71 | 19 | 41 |
| NK Cells % | 0.064 | -0.37 | 0.50 | 0.77 | 31 | 62 |
| Messenger RNA Interleukin-12p35 | -0.069 | -0.61 | 0.48 | 0.80 | 19 | 41 |
| CD4 T Cells % | -0.053 | -0.48 | 0.38 | 0.81 | 31 | 62 |
| Messenger RNA Interleukin-6 | -0.057 | -0.60 | 0.49 | 0.84 | 19 | 41 |
| Messenger RNA Tumor-Necrosis Factor Alpha | -0.050 | -0.59 | 0.49 | 0.86 | 19 | 41 |
| GDF15 (Growth Differentiation Factor 15 Level) | 0.055 | -0.60 | 0.71 | 0.87 | 11 | 52 |
| Messenger RNA Interleukin-27-p28 | -0.035 | -0.58 | 0.51 | 0.90 | 19 | 41 |
| Interleukin (IL)-23 | 0.029 | -0.51 | 0.57 | 0.92 | 19 | 41 |
| CD19 B Cells % | -0.017 | -0.45 | 0.41 | 0.94 | 31 | 62 |
| CD4 / CD8 T Cells | -0.011 | -0.44 | 0.42 | 0.96 | 31 | 62 |
| Mean Corpuscular Haemoglobin Concentration | -0.006 | -0.29 | 0.27 | 0.97 | 51 | 1105 |
| Direct Bilirubin | 0.000 | -0.43 | 0.43 | 1.00 | 31 | 62 |
| Interleukin (IL)-10 | 0.000 | -0.97 | 0.97 | 1.00 | 7 | 10 |

**Abbreviations:** IFN-$\gamma$: interferon-gamma SMD: standardised mean difference; LL: lower limit; POP: post-operative pneumonia; UL: upper limit.
