# Supplementary material for: Biomarkers associated with post-operative pneumonia: a systematic review and meta-analysis
Source: BMC Pulm Med. 2026 Jul 16;26:455. doi: 10.1186/s12890-026-04486-7 (PMC13651040; doi:10.1186/s12890-026-04486-7)
Supplement: Supplementary file 5 [file 12890_2026_4486_MOESM5_ESM.docx]

**Additional File 5.**

**Additional File 5 Table:** Post-operative biomarkers reported in 1 study only.

| Biomarker | SMD | Lower Limit | Upper Limit | *p* | Sample Size | |
| --- | --- | --- | --- | --- | --- | --- |
|  |  |  |  |  | POP | No-POP |
| Day 1 Post-Operative Biomarkers | | | | | | |
| Toll-like Receptor 4 | 1.014 | 0.75 | 1.28 | <0.0001 | 93 | 172 |
| Soluble myeloid triggering receptor (sTREM1) | 0.814 | 0.55 | 1.08 | <0.0001 | 93 | 172 |
| CD4 T Cells | -0.712 | -0.98 | -0.44 | <0.0001 | 61 | 576 |
| CD8 HLADR | 0.662 | 0.40 | 0.93 | <0.0001 | 61 | 576 |
| Albumin | -0.661 | -0.93 | -0.39 | <0.0001 | 61 | 576 |
| Serum Amyloid A | 0.609 | 0.35 | 0.87 | <0.0001 | 93 | 172 |
| CD4 CD45RA | -0.574 | -0.84 | -0.31 | <0.0001 | 61 | 576 |
| Alanine Transaminase | 0.428 | 0.16 | 0.69 | 0.002 | 61 | 576 |
| Endocan | 0.440 | -0.07 | 0.95 | 0.09 | 17 | 138 |
| CD4 CD28 | -0.196 | -0.46 | 0.07 | 0.15 | 61 | 576 |
| Messenger RNA IL-23-p19 | 0.322 | -0.23 | 0.87 | 0.25 | 19 | 41 |
| CD8 T Cells | 0.153 | -0.11 | 0.42 | 0.26 | 61 | 576 |
| Secretory Leucocyte Peptidase Inhibitor | -0.274 | -1.16 | 0.61 | 0.55 | 6 | 27 |
| Messenger RNA IL-10 | 0.165 | -0.38 | 0.71 | 0.55 | 19 | 41 |
| Messenger RNA IL-12p35 | 0.142 | -0.40 | 0.69 | 0.61 | 19 | 41 |
| Messenger RNA IL-6 | -0.075 | -0.62 | 0.47 | 0.79 | 19 | 41 |
| CD8 CD28 | -0.028 | -0.29 | 0.24 | 0.84 | 61 | 576 |
| IFN-$\boldsymbol{\gamma}$ | 0.027 | -0.52 | 0.57 | 0.92 | 19 | 41 |
| IL-23 | 0.022 | -0.52 | 0.57 | 0.94 | 19 | 41 |
| Messenger RNA interleukin-27-p28 | -0.019 | -0.56 | 0.52 | 0.94 | 19 | 41 |
| Neutrophil-to-Granulocyte Ratio | 0.010 | -0.30 | 0.32 | 0.95 | 44 | 358 |
| Messenger RNA IFN-$\boldsymbol{\gamma}$ | -0.009 | -0.55 | 0.54 | 0.98 | 19 | 41 |
| Messenger RNA TNF-$\boldsymbol{\alpha}$ | -0.006 | -0.55 | 0.54 | 0.98 | 19 | 41 |
| Day 2 Post-Operative Biomarkers | | | | | | |
| Neutrophil-to-Lymphocyte Ratio | 1.170 | 0.81 | 1.53 | <0.0001 | 49 | 114 |
| Systemic Immune Inflammation Index | 0.572 | 0.23 | 0.91 | 0.001 | 49 | 114 |
| Lymphocyte-to-Monocyte Ratio | -0.382 | -0.72 | -0.04 | 0.03 | 49 | 114 |
| Neutrophil-to-Granulocyte Ratio | 0.307 | -0.01 | 0.62 | 0.06 | 44 | 358 |
| Endocan | -0.433 | -0.94 | 0.07 | 0.09 | 17 | 138 |
| Platelet-to-Lymphocyte Ratio | 0.267 | -0.07 | 0.60 | 0.12 | 49 | 114 |
| Albumin | -0.244 | -0.58 | 0.09 | 0.15 | 49 | 114 |
| Secretory Leucocyte Peptidase Inhibitor | 0.642 | -0.26 | 1.54 | 0.16 | 6 | 27 |
| Hb | 0.014 | -0.32 | 0.35 | 0.93 | 49 | 114 |
| Day 3 Post-Operative Biomarkers | | | | | | |
| Neutrophil-to-Granulocyte Ratio | 0.353 | 0.04 | 0.67 | 0.03 | 44 | 358 |
| Secretory Leucocyte Peptidase Inhibitor | 0.658 | -0.24 | 1.56 | 0.15 | 6 | 27 |
| Endocan | -0.339 | -0.84 | 0.17 | 0.19 | 17 | 138 |
| Neutrophil-to-Granulocyte Ratio | 0.353 | 0.04 | 0.67 | 0.03 | 44 | 358 |
| Day 5 Post-Operative Biomarkers | | | | | | |
| Messenger RNA IFN-$\boldsymbol{\gamma}$ | -0.303 | -0.85 | 0.24 | 0.28 | 19 | 41 |
| Messenger RNA IL-10 | 0.153 | -0.39 | 0.70 | 0.58 | 19 | 41 |
| Messenger RNA TNF-$\boldsymbol{\alpha}$ | -0.106 | -0.65 | 0.44 | 0.70 | 19 | 41 |
| Messenger RNA IL-23-p19 | -0.054 | -0.60 | 0.49 | 0.85 | 19 | 41 |
| Messenger RNA IL-27-p28 | 0.054 | -0.49 | 0.60 | 0.85 | 19 | 41 |
| IFN-$\boldsymbol{\gamma}$ | -0.050 | -0.59 | 0.49 | 0.86 | 19 | 41 |
| IL-23 | 0.037 | -0.51 | 0.58 | 0.89 | 19 | 41 |
| Messenger RNA IL-6 | 0.035 | -0.51 | 0.58 | 0.90 | 19 | 41 |
| Messenger RNA IL-12p35 | 0.013 | -0.53 | 0.56 | 0.96 | 19 | 41 |
| Day 6 Post-Operative Biomarkers | | | | | | |
| CRP | 0.692 | 0.46 | 0.92 | <0.0001 | 96 | 343 |
| Day 7 Post-Operative Biomarkers | | | | | | |
| CRP | 0.794 | 0.56 | 1.03 | <0.0001 | 96 | 343 |
| PCT | 0.993 | 0.76 | 1.23 | <0.0001 | 96 | 343 |

**Note:** nil biomarker data for day 4 post-operative. **Abbreviations:** CRP = C-Reactive Protein; Hb = haemoglobin; IFN-$\gamma$ = interferon-gamma; IL = interleukin; mRNA = messenger ribonucleic acid; PCT = procalcitonin; POP = post-operative pneumonia; SMD = standardised mean difference; TNF-$\alpha$ = tumor necrosis factor-alpha; WBC = white blood cell count.
