# Supplementary material for: Biomarkers associated with post-operative pneumonia: a systematic review and meta-analysis
Source: BMC Pulm Med. 2026 Jul 16;26:455. doi: 10.1186/s12890-026-04486-7 (PMC13651040; doi:10.1186/s12890-026-04486-7)
Supplement: Supplementary file 6 [file 12890_2026_4486_MOESM6_ESM.docx]

Additional File 6

**Additional File 6 Table:** Risk of bias sensitivity analysis profile for pre- and post-operative biomarkers

| **Statistically Significant Biomarkers** | **Timepoint** | **K** | **I^2^ %** | **ROB Profile**  (Low, Some, High, Very High) | **Sensitivity Analysis**  **Feasible?** | **Effect after Sensitivity Analysis** | **Certainty**  **Level** |
| --- | --- | --- | --- | --- | --- | --- | --- |
| Platelet-to-neutrophil ratio | Pre-Op | 2 | 0.00 | Some = 2 | No | Low Study Numbers | N/A |
| Monocyte-to-lymphocyte ratio | Pre-Op | 2 | 0.00 | Some = 2 | No | Low Study Numbers | N/A |
| SII | Pre-Op | 5 | 77.20 | High = 4  Very High = 1 | No | Low Study Numbers | N/A |
| SIRI | Pre-Op | 2 | 63.64 | Some = 2 | No | Low Study Numbers | N/A |
| Blood urea nitrogen | Pre-Op | 4 | 56.06 | Some = 1; High = 1;  Very High = 2 | No | Low Study Numbers | N/A |
| Red blood cell count | Pre-Op | 2 | 35.33 | Some = 1  High = 1 | No | Low Study Numbers | N/A |
| Haemoglobin | Pre-Op | 10 | 61.69 | Some = 4; High = 4; Very High = 2 | Yes | Attenuated | Very Low |
| Albumin | Pre-Op | 9 | 91.44 | Some = 1; High = 5; Very High = 3 | No | N/A | N/A |
| Lymphocyte count | Pre-Op | 9 | 79.86 | Some = 2; High = 4; Very High = 3 | No | N/A | N/A |
| Procalcitonin (PCT) | Day 1 | 12 | 97.25 | Some = 6;  Very High = 6 | Yes | Attenuated | Very Low |
|  | Day 2 | 7 | 95.81 | Some = 3;  Very High = 4 | Yes | Attenuated | Very Low |
|  | Day 3 | 8 | 95.41 | Some = 4;  Very High = 4 | Yes | Attenuated | Very Low |
|  | Day 6 | 2 | 0.00 | Very High = 2 | No | Low Study Numbers | N/A |
| C-Reactive Protein (CRP) | Day 2 | 5 | 57.88 | Some = 3;  Very High = 2 | Yes | Attenuated | Very Low |
|  | Day 4 | 4 | 0.00 | Some = 3;  Very High = 1 | Yes | Robust | Low |
|  | Day 5 | 4 | 77.48 | Some = 3;  High = 1 | Yes | Robust | Very Low |
| Interleukin (IL)-6 | Day 3 | 2 | 69.39 | Some = 2 | No | Low Study Numbers | N/A |
|  | Day 4 | 2 | 61.83 | Some = 2 | No | Low Study Numbers | N/A |
| White blood cell count (WBC) | Day 3 | 5 | 97.07 | Some = 3;  Very High = 2 | Yes | Attenuated | Very Low |
|  | Day 4 | 4 | 0.00 | Some = 3;  Very High = 1 | Yes | Robust | Low |

Sensitivity analyses were conducted for statistically significant meta-analysed pre- and post-operative biomarkers, and where sufficient data were available. Sensitivity analyses were performed only when two or more studies remained following removal of studies with high or very high risk of bias. **Abbreviations:** CRP = C-Reactive Protein; Hb = haemoglobin; IL-6 = interleukin-6; PaC02 = partial pressure of carbon dioxide in arterial blood; Pa02 = partial pressure of oxygen in arterial blood; PCT = procalcitonin; ROB = risk of bias; SII = Systemic Immune Inflammation Index; SIRI = Systemic Inflammatory Response Index; WBC = white blood cells

**Sensitivity analysis for pre-operative biomarkers:**

**Additional File Figure 6.1.** Haemoglobin (risk of bias)

*Meta-analysis of pooled data for haemoglobin at pre-operative baseline, comparing pneumonia with no-pneumonia, excluding studies with high and very high risk of bias.*

**Additional File Figure 6.2.** Haemoglobin (data conversion)

*Meta-analysis of pooled data for haemoglobin at pre-operative baseline, comparing pneumonia with no-pneumonia, excluding studies requiring data conversion.*

**Additional File Figure 6.3.** Albumin

*Meta-analysis of pooled data for albumin at pre-operative baseline, comparing pneumonia with no-pneumonia, excluding studies requiring data conversion.*

**Additional File Figure 6.4.** Lymphocyte Count

*Meta-analysis of pooled data for lymphocyte count at pre-operative baseline, comparing pneumonia with no-pneumonia, excluding studies requiring data conversion.*

**Sensitivity analysis for post-operative biomarkers:**

**1) C-Reactive Protein (CRP):**

**Additional File Figure 6.5.** CRP at day two

*Meta-analysis of pooled data for CRP at day two post-operatively, comparing pneumonia with no-pneumonia, excluding studies with high and very high risk of bias.*

**Additional File Figure 6.6.** CRP at day four

*Meta-analysis of pooled data for CRP at day four post-operatively, comparing pneumonia with no-pneumonia, excluding studies with high and very high risk of bias.*

**Additional File Figure 6.7.** CRP at day five

*Meta-analysis of pooled data for CRP at day five post-operatively, comparing pneumonia with no-pneumonia, excluding studies with high and very high risk of bias.*

**2) White Blood Cell Count (WBC):**

**Additional File Figure 6.8.** WBC at day three

*Meta-analysis of pooled data for WBC at day three post-operatively, comparing pneumonia with no-pneumonia, excluding studies with high and very high risk of bias.*

**Additional File Figure 6.9.** WBC at day four

*Meta-analysis of pooled data for WBC at day four post-operatively, comparing pneumonia with no-pneumonia, excluding studies with high and very high risk of bias.*

**3) Procalcitonin (PCT):**

**Additional File Figure 6.10.** PCT at day one

*Meta-analysis of pooled data for PCT at day one post-operatively, comparing pneumonia with no-pneumonia, excluding studies with high and very high risk of bias.*

**Additional File Figure 6.11.** PCT at day two

*Meta-analysis of pooled data for PCT at day two post-operatively, comparing pneumonia with no-pneumonia, excluding studies with high and very high risk of bias.*

**Additional File Figure 6.12.** PCT at day three

*Meta-analysis of pooled data for PCT at day three post-operatively, comparing pneumonia with no-pneumonia, excluding studies with high and very high risk of bias.*
